# Influence of Surface Chemistry on Metal Deposition Outcomes in Copper Selenide-Based Nanoheterostructure Synthesis

**DOI:** 10.1021/acs.langmuir.4c01817

**Published:** 2024-07-27

**Authors:** Riti Sen, Shelby L. Millheim, Tyler M. Gordon, Jill E. Millstone

**Affiliations:** † Department of Chemistry, University of Pittsburgh, 219 Parkman Avenue, Pittsburgh, Pennsylvania 15260, United States; ‡ Department of Chemical and Petroleum Engineering, University of Pittsburgh, 3700 O’Hara Street, Pittsburgh, Pennsylvania 15261, United States; § Department of Mechanical Engineering and Materials Science, University of Pittsburgh, 3700 O’Hara Street, Pittsburgh, Pennsylvania 15261, United States

## Abstract

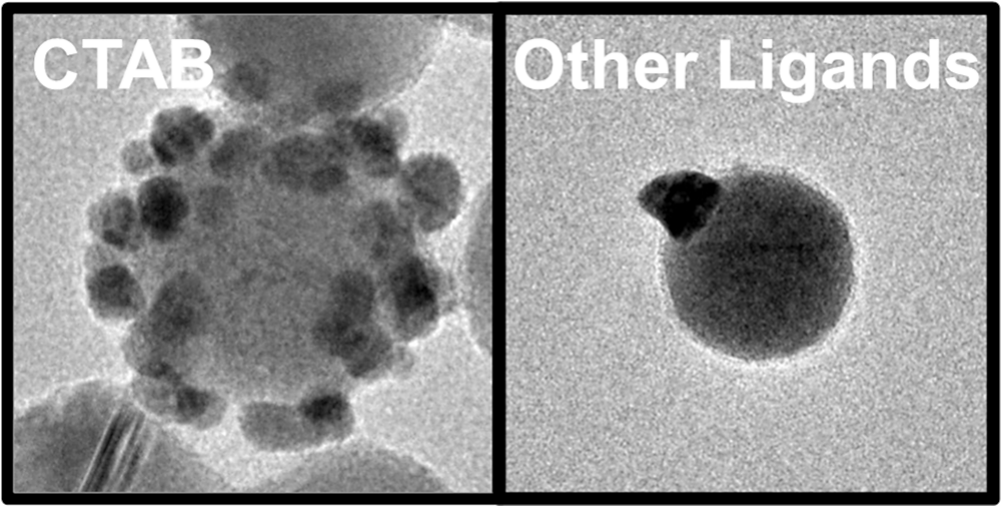

The use of nanoparticle surface chemistry to direct metal deposition has been well-studied in the modification of metal nanoparticle substrates but is not yet well-established for metal chalcogenide particle substrates, although integration of these particles into nanoheterostructures is of high interest. In this report, we investigate the effect of Cu_2–*x*_Se surface chemistry on the morphology of metal deposition on these plasmonic semiconductor nanoparticles. Specifically, we functionalize Cu_2–*x*_Se nanoparticles with a suite of 12 different ligands and investigate how different aspects of the ligand structure do or do not impact the morphology and extent of subsequent metal deposition on the Cu_2–*x*_Se surface. Surprisingly, our results indicate that the morphology of the resulting metal deposits and the extent of metal deposition onto the existing Cu_2–*x*_Se particle substrate are indistinguishable for the majority of ligands tested. An exception to these findings is observed for particles functionalized by quaternary alkylammonium bromides, which exhibit statistically distinct metal deposition patterns compared to all other ligands tested. We hypothesize that this unique behavior is due to a cooperative binding mechanism of the quaternary alkylammonium bromides to the surface of copper selenide. Taken together, these results yield both new strategies for controlling postsynthetic modification of copper selenide nanoparticles and also reveal limitations of surface chemistry-based approaches for this system.

## Introduction

There is a large and growing interest in the creation of metal–semiconductor hybrid nanomaterials because of their unique properties and applications including in photocatalysis,^1−5^ optoelectronics,^6,7^ and biological imaging/diagnostics.^8−10^ To efficiently match structure with function, there has been sustained interest in establishing synthetic methods for precise control of their morphology.^11−15^ A particularly successful approach for tuning these heterostructures has been postsynthetic modification of starting nanoparticle (NP) substrates.^16−22^ There are several advantages to postsynthetic modification strategies for tuning nanoheterostructure morphology including the improved ability to tailor interfaces within the structure such as interface size, composition, number, and/or extent (e.g., gradient). Strategies such as cation/anion exchange and galvanic replacement have led to remarkably complex structures,^16,17,19,23−28^ with both established and still studied new physical and chemical behaviors of interest.^29−34^

Another mode for generating nanoheterostructures via postsynthetic modification is to leverage the molecular surface chemistry of the starting NP substrate.^35−41^ In this approach, one may use NP surface chemistry to modulate the flux of the precursor to the NP surface and block, slow, and/or facilitate growth at particular sites. The use of NP surface chemistry to direct metal deposition has been well-studied in the modification of metal NP substrates^20,42−47^ but is not yet established for metal chalcogenide particle substrates, although these particles are of high interest for incorporation into nanoheterostructures.

Here, we investigate the effects of Cu_2–*x*_Se NP surface chemistry on postsynthetic metal deposition using metals that are known to deposit on these structures as opposed to cation exchange with them: Au and Pt.^18,21^ We chose Cu_2–x_Se NPs as our starting material because they are readily synthesized in a variety of sizes using an aqueous, ambient approach, and their plasmonic properties make them useful candidates for downstream applications. The aqueous route is particularly attractive because it not only eliminates less sustainable solvents but also takes place in air, which enhances the plasmonic properties of the Cu_2–*x*_Se that arise from hole formation via Cu oxidation.^48−51^ Cu_2–*x*_Se NPs also exhibit an isotropic crystal structure, which simplifies interpretation of the heterostructure formation mechanisms by displaying uniform crystal facets. In total, we analyze the morphology of metal deposits on 12 different Cu_2–*x*_Se surface chemistries, which survey a variety of ligand charges, molecular weights, and surface–ligand binding moieties. Using this suite of conjugates, we consider multiple factors that may influence the interaction and addition of metal ion precursors to the Cu_2–*x*_Se surface, including surface charge, surface ligand density, and ligand shell stability. Surprisingly, we find that the metal deposition morphology is indistinguishable between the majority of the ligands tested, except for the quaternary alkylammonium bromides, suggesting that metal deposition on these surfaces is remarkably insensitive to ligand chemistry.

## Experimental Section

### General Methods and Materials

Copper(II) sulfate pentahydrate (CuSO_4_·5 H_2_O, >98%), selenium dioxide (SeO_2_, >99.9%), hexadecyltrimethylammonium bromide (CTAB, 99%), trimethyloctyl-ammonium bromide (TMOAB, 98%), tetraoctylammonium bromide (TOAB, 98%), poly(vinylpyrrolidone) (PVP, MW: 55,000 Da), sodium dodecyl sulfate (SDS, >99%), hydrogen tetrachloroaurate trihydrate (HAuCl_4_·3 H_2_O, 99.999%), l-ascorbic acid (99%), potassium tetrachloroplatinate(II) (K_2_PtCl_4_, 98%), and sodium hydroxide (NaOH, 98%) were obtained from Sigma-Aldrich (St. Louis, MO) and used as received. Poly(vinylpyrrolidone) (PVP, MW:3500 Da, 10,000 Da) was purchased from Thermo Scientific (Fair Lawn, NJ) and used as received. 11-Mercaptoundecanoic acid (MUA, 98%), 12-mercaptododecanoic acid (MDA, 98%), and 3-mercaptobenzoic acid (MBA) were purchased from Santa Cruz Biotechnologies, Inc. (Dallas, TX) and used as received. Poly(ethylene glycol) methyl ether thiol (PEGSH, MW:1000 Da, 5000 Da) was purchased from Laysan Bio Inc. (Arab, AL) and used as received. NANOpure water (Thermo Scientific, >18.2 MΩ·cm) was used for all washing, synthesis, and purification protocols as well as in the preparation of all solutions, unless otherwise noted. All stock solutions were aqueous and prepared fresh before each reaction, unless otherwise noted. All glassware was washed with aqua regia (3:1 ratio of concentrated HCl and HNO_3_ by volume) and rinsed thoroughly with water. **Caution**: *Aqua regia is highly toxic and corrosive and requires personal protective equipment. Aqua regia should be handled in a fume hood only.*

### Synthesis of CTAB-Capped Cu_2–*x*_Se NPs

Cu_2–*x*_Se NPs were synthesized by using a room temperature, seed-mediated procedure under ambient conditions. First, 10 mL of 0.7 mM CTAB was prepared in a scintillation vial on a thermomixer (Eppendorf R Thermomixer) at a speed of 350 rpm. 500 μL of 0.25 M SeO_2_ was added to the CTAB solution, followed by a rapid injection of 200 μL of 1.14 M aqueous ascorbic acid solution to promote the nucleation of selenium seeds, as indicated by a color change of the solution from clear to bright orange. After mixing for 10 min, 500 μL of 0.50 M CuSO_4_ was added to the orange Se seed solution, followed by another quick injection of 300 μL of 1.14 M ascorbic acid. After 10 s, the solution turned dark brown. This brown solution was allowed to mix for approximately 15 h at room temperature during which the solution turned dark green. The Cu_2–*x*_Se NP product was then purified via centrifugation as follows. First, the as-synthesized NPs were transferred to a 50 mL Falcon tube. 5 mL of 0.7 mM CTAB solution was added to the Falcon tube and diluted to the 50 mL mark with water. The NP solutions were centrifuged in an Eppendorf 5804 centrifuge with a fixed angle rotor (FA-45-6-30) (Eppendorf, Inc., Hauppauge, NY) at a force of 16,639 rcf at 25 °C for 10 min. The resulting supernatant was carefully removed, and the soft NP pellet was resuspended in approximately 50 mL of H_2_O for additional centrifugation. This washing procedure was repeated two additional times.

### Ligand Exchange of CTAB-Cu_2–*x*_Se NPs

To exchange CTAB on the Cu_2–*x*_Se NPs with a ligand of interest, 2 mL of purified CTAB-capped Cu_2–*x*_Se with an O.D. of 2.0 a.u. at λ_max_, was added to a scintillation vial. To this vial, 18 mL of a specific concentration of ligand solution was added. The specific ligand concentrations were chosen to ensure vast excess of the secondary ligands in solution relative to the available NP surface area (Table 1) (see Supporting Information for additional details). The reaction solution was allowed to mix at 350 rpm (Eppendorf R Thermomixer) and equilibrate at room temperature for 8 h, after which the particles were purified through centrifugation as follows. The NP solutions were centrifuged in an Eppendorf 5804 centrifuge with a fixed angle rotor (FA-45-6-30) (Eppendorf, Inc., Hauppauge, NY) at a force of 16,639 rcf at 25 °C for 10 min. The resulting supernatant was carefully removed, and the soft NP pellet was resuspended in approximately 50 mL of H_2_O for additional centrifugation. This washing procedure was repeated three additional times.

**Table 1:** Secondary Ligand Concentrations for Ligand Exchange Procedure

**ligand identity**	concentration (mM)
poly(vinylpyrrolidone) (MW: 3.5 kDa, 10 kDa)	15
poly(vinylpyrrolidone) (MW: 55 kDa)	0.2
poly(ethylene glycol) methyl ether thiol (MW: 1 kDa, 5 kDa)	15
sodium dodecyl sulfate	90
trimethyloctylammonium bromide	2.1
tetraoctylammonium bromide	2.1
11-mercaptoundecanoic acid	0.1 in 20 mM NaOH
12-mercaptododecanoic acid	0.1 in 20 mM NaOH
3-mercaptobenzoic acid	0.1 in 20 mM NaOH

### Au Deposition on Cu_2–*x*_Se NPs

To deposit Au on the Cu_2–*x*_Se NPs (as-synthesized or ligand exchanged), a 1 mL solution of purified Cu_2–*x*_Se NPs with an O.D. of 2 a.u. at λ_max_ (cuvette path length = 1 cm) was prepared in a 1.5 mL Eppendorf tube. To the Eppendorf tube, 40 μL of a 2 mM aqueous ascorbic acid solution was injected, immediately followed by vortexing for 10 s. To this mixture was added 40 μL of a 2 mM aqueous HAuCl_4_ solution, followed by short vortexing, after which the solution turned a green-gray color. This green-gray solution was shaken on a thermomixer (Eppendorf R Thermomixer) at 1000 rpm at room temperature for 8 h, after which the solution was purified through centrifugation as follows. The NP solutions were centrifuged in an Eppendorf 5424 centrifuge with a fixed angle rotor (FA-45-24-11) (Eppendorf, Inc., Hauppauge, NY) at 15,000 rpm at 25 °C for 5 min. After centrifugation, a small pellet remained, and the clear supernatant was removed. The Eppendorf tube was filled to 1 mL with water, and the Cu_2–*x*_Se NPs were resuspended. This washing procedure was repeated two additional times.

### Sequential Au Deposition on Cu_2–*x*_Se NPs

For the sequential deposition of Au on Cu_2–*x*_Se NPs (as-synthesized or ligand exchanged), the same postsynthetic modification procedure as above was used. The remaining pellets of the purified Au–Cu_2–*x*_Se NPs were resuspended in 1 mL of water in an Eppendorf tube. To the Eppendorf tube, 40 μL of 2 mM ascorbic acid was injected, followed by vortexing for 10 s. To the Eppendorf tube was added 40 μL of a 2 mM HAuCl_4_ solution, followed by vortexing. This solution was shaken on a thermomixer at 1000 rpm at room temperature for 8 h, after which the solution was purified through centrifugation as follows (and also described above). The NP solutions were centrifuged in an Eppendorf 5424 centrifuge with a fixed angle rotor (FA-45-24-11) (Eppendorf, Inc., Hauppauge, NY) at 15,000 rpm at 25 °C for 5 min. After centrifugation, a small pellet remained, and the clear supernatant was removed. The Eppendorf tube was filled to 1 mL with water, and the Cu_2–*x*_Se NPs were resuspended. This washing procedure was repeated two additional times. For the third and fourth metal depositions, a 1 mM ascorbic acid and 1 mM HAuCl_4_ solution were used.

### Transmission Electron Microscopy (TEM)

All samples were prepared for TEM by drop casting an aliquot of purified solution (diluted 1:100 with water) onto carbon-film-coated 200 mesh Cu TEM grids (Ted Pella, Inc., Redding, CA), allowed to dry under ambient conditions, and stored under vacuum prior to TEM analysis. A Hitachi H-9500 TEM at 300 kV was used for all imaging (Nanoscale Fabrication and Characterization Facility (NFCF), Petersen Institute of Nanoscience and Engineering (PINSE), University of Pittsburgh). Images were analyzed using Digital Micrograph v2.10.1282.0 (Gatan, Inc.) or ImageJ v 1.47d (National Institutes of Health, USA) software.

### Powder X-ray Diffraction (PXRD)

Purified samples of Cu_2–*x*_Se NPs and Au–Cu_2–*x*_Se NPs were lyophilized by using a LabConoco Freezone 6 lyophilizer to obtain dry powders. The NP powders were packed in 0.50 mm capillary tubes (Hampton Research, Aliso Viejo, CA) and flame-sealed. PXRD patterns were collected on a Bruker X8 Prospector Ultra (Department of Chemistry, University of Pittsburgh) at 45 kV, 0.65 mA equipped with a IμS microfocus Cu Kα X-ray source (λ = 1.54178 Å) with a scan speed of 0.5 s/step from 12.00 to 108.00° with a step size of 0.02°. All spectra were baseline corrected with respect to the spectrum of the amorphous glass background.

### Ultraviolet–Visible-Near Infrared (UV–vis–NIR) Extinction Spectroscopy

The extinction spectra of Cu_2–*x*_Se NPs were collected by using a Cary 5000 UV–vis–NIR spectrophotometer (Agilent, Inc., Santa Clara, CA). Purified NPs were suspended in 1 mL of H_2_O and placed in 1 cm quartz cuvettes (Hellma, Inc., Plainview, NY). The spectra were recorded from 1350 to 300 nm at a scanning rate of 1010 nm/min, with a grating and detector changeover occurring at 800 nm and a source changeover occurring outside the scanned range at 290 nm. Spectra were baseline corrected with respect to 1 mL of H_2_O.

### Zeta Potential Characterization

Zeta potential measurements were taken on an Anton Paar Litesizer 500, at 25 °C, and the Smoluchowski approximation was used to analyze the data. To a clean, dust-free, Omega glass cuvette, 10–100 μL of NP solution was added and diluted to 1 mL with water and then filtered using a 0.45 μm poly(propylene) membrane syringe filter. For each NP sample, multiple NP concentrations were measured to assess any convolution from multiple scattering events.

### X-ray Photoelectron Spectroscopy (XPS) Characterization

Samples were prepared by drop casting an aliquot of purified NPs onto p-doped (boron) silicon wafers (University Wafer, Boston, MA) that were cleaned for ultrahigh vacuum analysis. XPS spectra were obtained using an ESCALAB 250XI XPS with a monochromated, microfocused Al Kα X-ray source (Materials Characterization Laboratory, Department of Chemistry, University of Pittsburgh, PA) at a spot size of 500 μm. All samples were sputtered using Ar^+^ at a beam energy of 4000 eV for ∼45 s prior to spectral collection to remove surface impurities. Survey and high-resolution spectra were collected with a pass energy of 150 and 50 eV and a step size of 1.0 and 0.1 eV, respectively. All spectra were charge referenced to adventitious carbon (284.8 eV) and fit using Thermo Scientific Advantage software. All spectra were averaged across at least three experimental replicates.

## Results and Discussions

### Results

As part of the formation process, metal deposition onto the surface of an existing NP substrate fundamentally requires an incoming metal atom (either as an ion or previously reduced) to both collide with and then add to the NP substrate. Several features of the NP substrate ligand shell may influence those collision and addition events, and we first outline these considerations as a background for the experiments and analysis that follows.

First, the dissociation of the pendant ligands may depend on the solubility of the ligand in the reaction environment, the strength of the binding interaction between the NP surface and a specific functional group(s) in the ligand, and intermolecular interactions between ligands. The dissociation of ligands from the surface can be quantitatively described by an equilibrium adsorption constant, *K*.^52,53^ Access to the NP surface also depends on the density of ligands covering it and can be quantified by the number of ligands per unit NP surface area (e.g., ligands/nm^2^) when treating small molecule ligands.^54,55^ For polymeric ligands, because there are multiple surface–ligand interactions per polymer chain, but may be few polymer chains per NP, polymeric ligand densities are not necessarily well-described by a metric such as ligands/nm^2^ and may also be described by shell thickness (as measured by DLS or TEM in some cases) or total ligand shell mass.^56,57^ The ability of atoms to diffuse and add to the NP surface may also be influenced by the overall charge of the ligand shell (or charges within it), which may attract, sequester, or repel incoming growth species. Ligand charge may be quantified as a bulk parameter of the colloid by measuring particle zeta potential.^52^

**Scheme 1:**
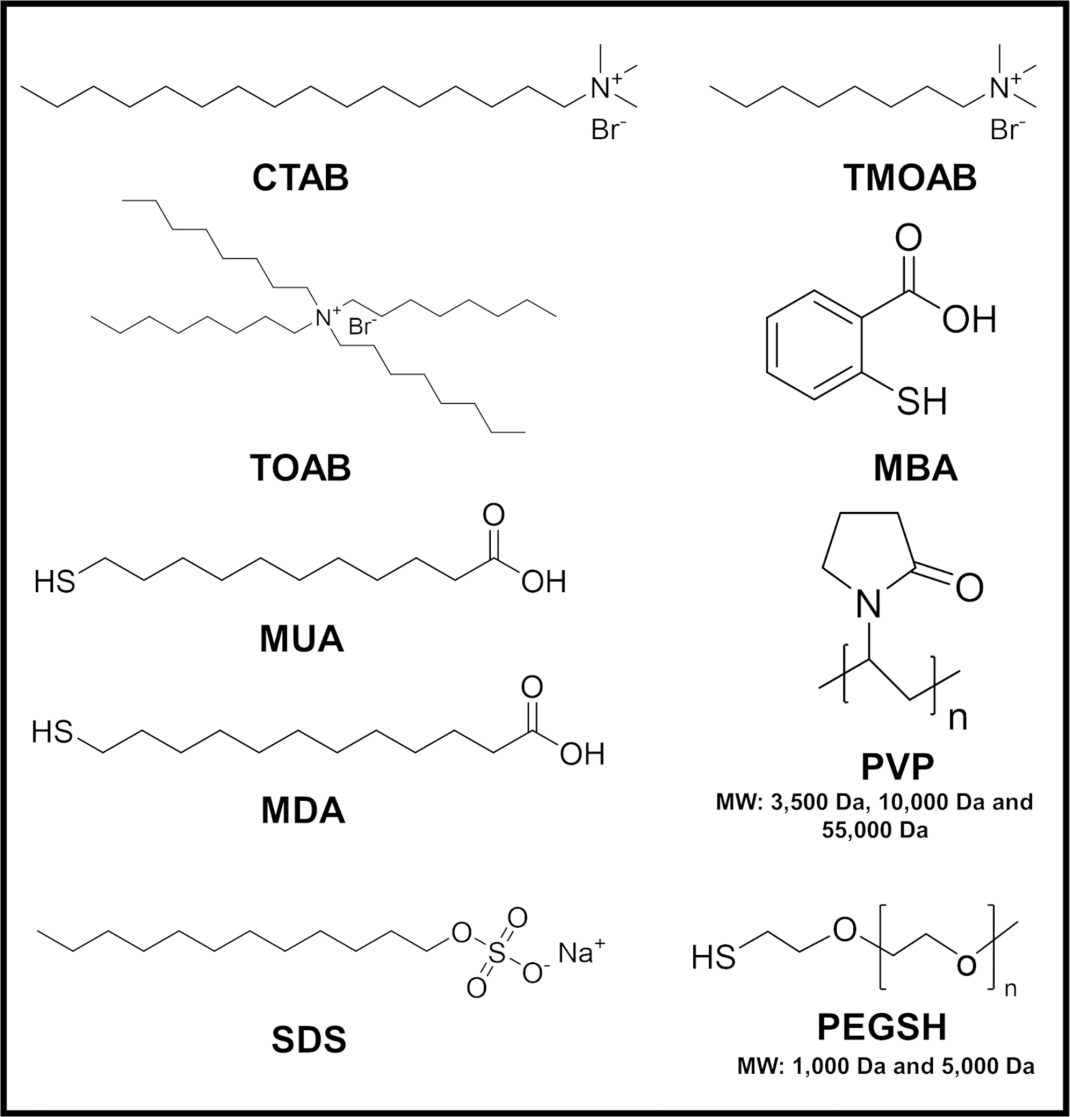
Schematic Representation of Ligands Used to Functionalize Colloidal Cu_2–*x*_Se NPs Hexadecyltrimethylammonium bromide (CTAB), trimethyloctyl-ammonium bromide (TMOAB), tetraoctylammonium bromide (TOAB), 11-mercaptoundecanoic acid (MUA), 12-mercaptododecanoic acid (MDA), 3-mercaptobenzoic acid (MBA), sodium dodecyl sulfate (SDS), poly(vinylpyrrolidone) (PVP, MW: 3500; 10,000; 55,000 Da), poly(ethylene glycol) methyl ether thiol (PEGSH, MW: 1000, 5000 Da).

In this report, we used a suite of 12 ligands (Scheme 1) with different particle binding groups, charges, sizes, and structures to explore each of these factors. Ligand choices were motivated by an effort to highlight these ligand considerations individually (although it is not possible to decouple them completely), as well as their solubility in water and their ability to colloidally stabilize the Cu_2–*x*_Se NPs in water (either as-synthesized or via ligand exchange). To perform a systematic study of the ligand impact on the resulting metal deposition (MD), we began with four ligands that survey a variety of ligand properties including charge, size, and particle binding moiety: CTAB, 3.5 kDa PVP, 1 kDa PEGSH, and SDS. Remaining ligand trials were then used to confirm and/or clarify results obtained from these four ligands.

All initial MD syntheses were tested using Au as the depositing metal and subsequently repeated using Pt. In all cases, we saw that ligand impacts on MD outcomes did not vary between Au and Pt (vide infra, Figure S8). For this report, we primarily discuss the Au deposition results because of two factors: (1) Pt deposits in a dendritic morphology on Cu_2–*x*_Se and therefore Au deposition is more straightforward to quantify in terms of number, dimensions, and volume of the deposits; (2) Au is a plasmonic material, and we were interested in the structural development and synthesis of Au–Cu_2–*x*_Se dual plasmonic nanoheterostructures.

In a typical experiment, we start with water-dispersible Cu_2–*x*_Se NPs (diameter = 45 ± 4 nm), capped with CTAB. To have a consistent core topology and size for all surface chemistries tested, we used mass action ligand exchange to remove CTAB from the Cu_2–*x*_Se cores and replace it with either 3.5 kDa PVP, 1 kDa PEGSH, or SDS ligands (Figure S7). The extent of the ligand exchange was investigated by several methods. Expected changes in NP zeta potentials indicate ligand exchange, and shifts in the Cu_2–*x*_Se localized surface plasmon resonance (LSPR) are also consistent with changing surface chemistry although not chemo-specific (Table S1, Figure S1). Analysis of XPS spectra of each exchanged particle indicates the removal of CTAB, the absence of bromide, and the presence of new carbon-containing species (vide infra and Figures S3–S6). PXRD patterns and size distribution histograms of the resulting NP-ligand conjugates indicate that NP size and crystallinity are preserved during the exchange process (Figure S2). Techniques such as ^1^H NMR quantification were not possible with the Cu_2–*x*_Se NPs because of the presence of paramagnetic Cu(II) ions.^58,59^ (See the Supporting Information for additional details on the ligand exchange characterization approach.)

**Figure 1:**
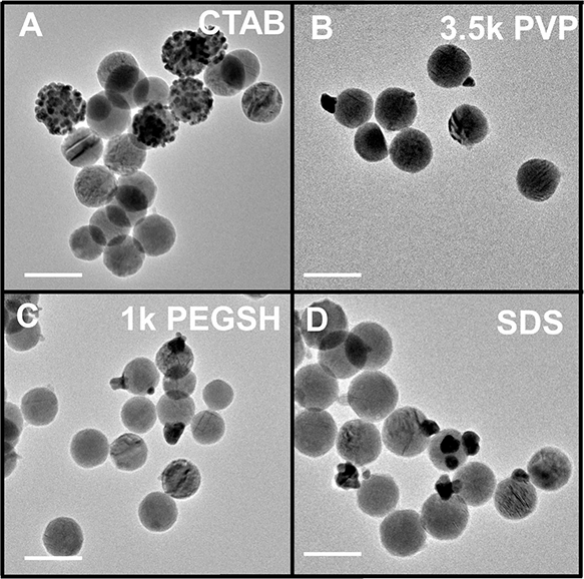
Representative TEM images of Au deposition on Cu_2–*x*_Se capped with (A) CTAB, (B) 3.5 kDa PVP, (C) 1 kDa PEGSH, and (D) SDS. Scale bars are 100 nm.

Reduction of HAuCl_4_ with ascorbic acid (1:1 molar ratio), in the presence of the Cu_2–*x*_Se NPs, under ambient conditions, resulted in the deposition of Au nanoislands on the surface of the Cu_2–*x*_Se NPs for all four ligand chemistries (Figure 1). This islandic (as opposed to uniform shell) deposition pattern may be expected from the high Au–Cu_2–*x*_Se lattice mismatch (∼27%) and is consistent with previous observations of Volmer–Weber growth patterns of Au on berzelianite Cu_2–*x*_Se.^60^ Deposition on CTAB-coated particles shows an isotropic distribution of multiple Au islands on each modified Cu_2–*x*_Se NP, where the islands roughly decorate the entire particle surface (Figure 1A). For Cu_2–*x*_Se NPs functionalized either with 3.5 kDa PVP, 1 kDa PEGSH, or SDS, NPs exhibit single island deposits as the dominant products, and at first inspection, there appears to be little difference in deposition patterns among the three NP types. This insensitivity to the ligand shell is surprising, since these ligands not only differ from each other in terms of structure and size but also differ in how they bind to the surface of Cu_2–*x*_Se, the charge around the ligand shell (as indicated by zeta potential measurements), as well as the intermolecular forces present within the ligand layer and at the surface of Cu_2–*x*_Se.

In order to better discern any differences in MD motifs observed on Cu_2–*x*_Se NPs with differing surface chemistries, we analyzed several aspects of the resulting nanoheterostructures including: (i) the number of particles exhibiting single vs multiple islands; (ii) the size of each island; (iii) the percentage of Cu_2–*x*_Se NPs that are modified by any deposit; and (iv) the percent of each NP surface area that is covered by the deposited metal.

### Number and Size of Au Islands on Cu_2–*x*_Se NPs

**Figure 2:**
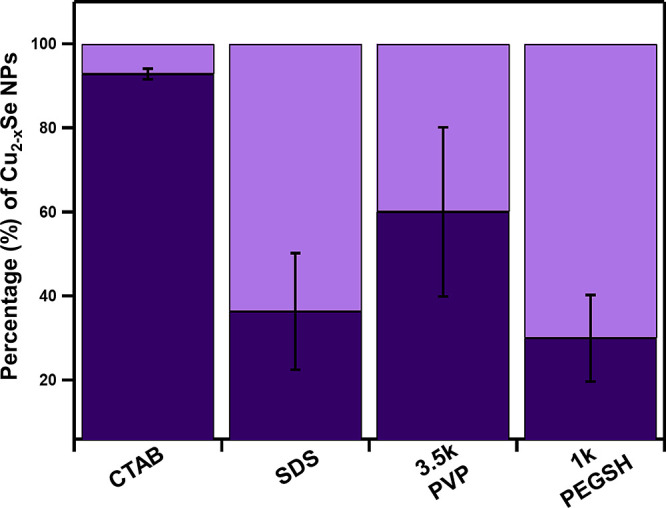
Statistical analysis of single (light purple) vs multiple islandic (dark purple) deposits on CTAB, 3.5 kDa PVP, 1 kDa PEGSH, and SDS-capped Cu_2–*x*_Se NPs. ANOVA analysis indicates that the CTAB population is statistically distinct from the other three ligands (Table S2).

The CTAB-capped Cu_2–*x*_Se NPs that underwent MD showed isotropic coatings of multiple island deposits (∼95%) (Figure 2). On the other hand, SDS-, 1 kDa PEGSH-, and 3.5 kDa PVP-terminated NPs exhibit anisotropic island deposition, where each Cu_2–*x*_Se particle has either one or two island deposits, but in no cases is an isotropic coating of islands observed as it is in the case of CTAB-capped Cu_2–*x*_Se NPs (Figure 2).

The average dimensions of each island are consistent across all four surface chemistries, where although the averages vary slightly, they are within the statistical error of one another (Table S3, CTAB = 10 ± 3 nm, 3.5 kDa PVP = 13 ± 7 nm, 1 kDa PEGSH = 14 ± 5 nm, and SDS = 15 ± 4 nm).

### Extent of Metal Deposition

**Figure 3:**
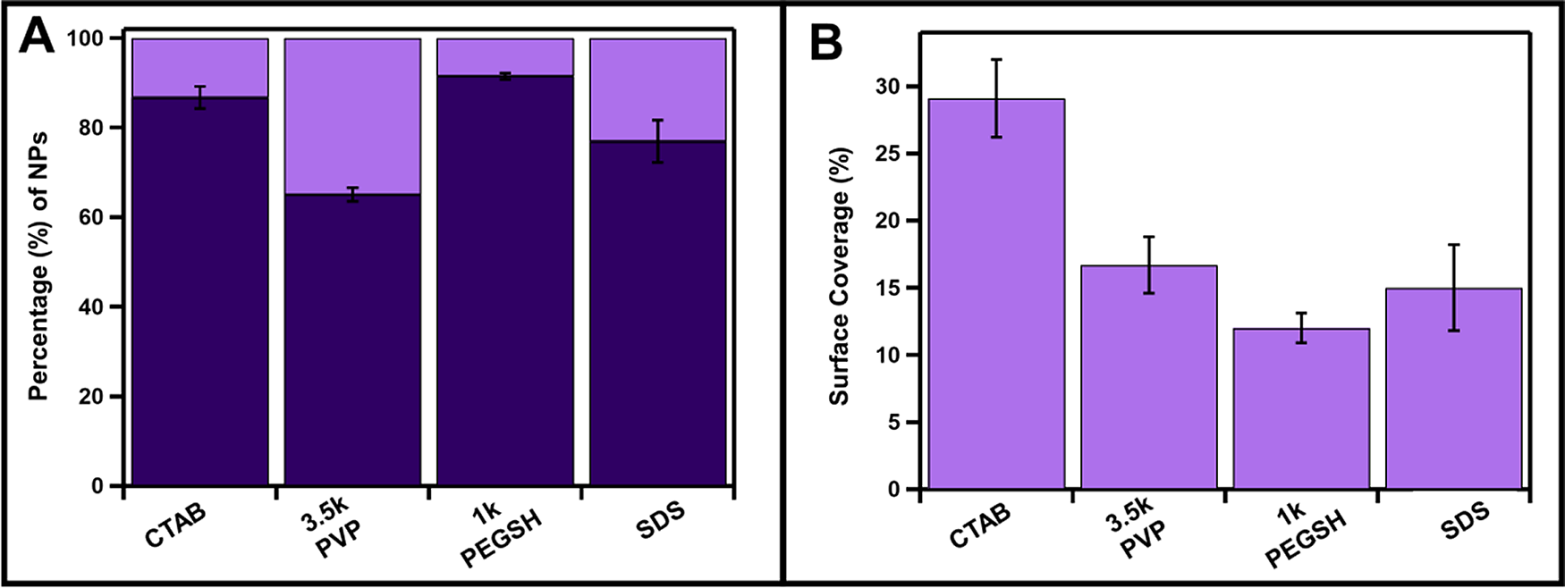
Percent modification (A) and percent surface coverage (B) of Au metal deposition on Cu_2–*x*_Se NPs capped with CTAB, 3.5 kDa PVP, 1 kDa PEGSH, and SDS. Dark purple represents the Cu_2–*x*_Se NPs that remain unchanged after metal deposition, and light purple represents the particles that undergo metal deposition.

The next aspect of the MD morphology we analyzed was the extent of metal deposition on Cu_2–*x*_Se as a function of different capping ligands. Here, we analyzed the extent of metal deposition as it relates to both the particle population as a whole, as well as each particle individually. For the population as a whole, we quantify the percentage of the Cu_2–*x*_Se NPs that underwent metal deposition in any form (% modification). To assess the extent of MD with respect to each particle, we measured the percentage of an individual Cu_2–*x*_Se NP surface that is covered by a Au deposit (% surface coverage) (Figure 3, Table 2). Across all four ligands, the percentage of Cu_2–*x*_Se NPs that undergo metal deposition is low (<35%) (Figure 3A). Specifically, PEGSH- and CTAB-capped NPs (∼10–15% modified particles) undergo MD less than NPs capped with PVP (∼35%) and SDS (∼30%). At the individual particle level, percent surface coverages are consistent with the dominant MD morphologies observed [i.e., high per particle for isotropic MD (CTAB) and comparatively low per particle for single/anisotropic metal deposits (PEGSH, PVP, and SDS)].

**Table 2:** Size of the Au Deposits, Percent Modification, and Percent Surface Coverage for the Various Capping Ligands on Cu_2–*x*_Se

**ligand type**	**size of Au deposit (nm)**	**percent modification (%)**	**percent surface coverage (%)**
CTAB	10 ± 3	13.3 ± 2.5	29.1 ± 2.9
3.5 kDa PVP	13 ± 7	35.0 ± 1.5	16.7 ± 2.1
1 kDa PEGSH	14 ± 5	8.5 ± 0.7	12.0 ± 1.1
SDS	15 ± 4	23.1 ± 4.7	15.0 ± 3.2

To estimate an average per particle surface coverage, we first quantified the amount of the Cu_2–*x*_Se NP surface area covered by a single Au island deposit. Here, we model each Au island as a hemisphere and assume that the length of the Au island is the radius of its base (see SI for detailed methodology). We then counted the average number of deposits on each modified Cu_2–*x*_Se NP. The average number of Au islands of each modified Cu_2–*x*_Se NP multiplied by the average surface area of its base gives the surface of a modified Cu_2–*x*_Se NP covered by the Au deposit. Then, we take the average surface area of a modified Cu_2–*x*_Se NP that is covered by Au and divide it by the total surface area of an average Cu_2–*x*_Se NP (derived from TEM size distributions) to obtain fractional surface coverage. Using this methodology, CTAB-capped Au–Cu_2–*x*_Se NPs show almost double the NP surface coverage (∼30%) of the other three ligands (∼12–15%) (Figure 3B), and this distinction is consistent with the isotropic Au MD observed exclusively on CTAB-terminated Cu_2–*x*_Se NPs.

## Discussion

As mentioned above, there are many mechanisms by which one can envision NP-ligand chemistry tuning the subsequent growth of metal island deposits. Below, we consider how our results comment on the effects of ligand charge, ligand density, and ligand adsorption constants on the resulting MD motifs on Cu_2–*x*_Se NPs and explore additional ligand chemistries to augment questions raised by these initial data.

First, we consider the impacts of ligand charge on MD processes. One may anticipate that the charge on the surface of Cu_2–*x*_Se NPs introduced by different ligand chemistries may impact the kinetics of metal precursor interactions with the NP surface and therefore the extent and/or morphology of the growing metal deposits. Depending on the charge of the metal precursor species (e.g., whether it is an anionic coordination complex such as [AuCl_4_]^−^ or a partially reduced Au precursor species), one may predict different outcomes based on the charge of the pendant Cu_2–*x*_Se ligands. For example, if the precursor species is anionic, then a negatively charged ligand shell may lead to either less deposition overall and/or fewer deposits per particle due to repulsive Coulombic interactions. However, our statistical analyses show no difference or trend in either the percent modification or surface coverage between SDS-functionalized Cu_2–*x*_Se NPs (negatively charged, zeta potential = −36.5 ± 1.3 mV) and those functionalized by neutral ligands (PVP (2.1 ± 0.6 mV) and PEGSH (7.0 ± 1.0 mV)) (*N.B.* |10 mV| is considered neutral^61^). This insensitivity of the MD morphology to the ligand shell charge suggests that the active metal precursor may not be a charged complex.

However, even if the reduction of the Au^3+^ precursor occurs before diffusion through the ligand shell, we may still expect to see an effect of the ligand shell thickness on the deposition morphology, where diffusion of the Au precursor to the surface of the NP would be slower for a bulkier surface ligand, leading to smaller and/or fewer islands.^62^ To test this hypothesis, with minimal convoluting factors, we also ligand exchanged CTAB-capped Cu_2–*x*_Se NPs with higher molecular weights of PVP (10 and 55 kDa) and PEGSH (5 kDa) and compare these new conjugates with their lower molecular weight counterparts (Figures S24–S26). For all molecular weights of the polymer ligands tested, we observe Au islands sizes, numbers, and modification fractions that are within error of one another (Figures S19 and S22, Table S3). These results suggest that diffusion of the Au precursor through these ligand shells is a fast step that does not measurably impact MD outcomes.

Next, we analyze our results in the context of the estimated ligand adsorption behavior. XPS analyses suggest that CTAB binds to the Cu_2–*x*_Se surface through the positively charged ammonium group (most likely coordinating to Se^4–^ anions on the surface), and we do not observe evidence of any associated halides at the surface (Figure S3). XPS of PEGSH-terminated Cu_2–*x*_Se NPs indicate that PEGSH is coordinated to the surface via Cu(II)–S interactions (Figure S4). For PVP-capped NPs, the N 1s spectra show only one binding energy corresponding to a free C–N species, whereas the O 1s spectra show two binding interactions corresponding to free C=O species and an O–Cu interaction at higher binding energy (Figure S5). Taken together, these data suggest that PVP interacts with the Cu_2–*x*_Se surface through the O (of the C=O). A similar interaction of PVP with Cu_2–*x*_Se thin films has also been reported in other works.^63,64^ XPS spectra of SDS-functionalized Cu_2–*x*_Se NPs do not reveal clear evidence of either sulfur or oxygen-based Cu(II) interactions but also did not show evidence of residual CTAB (Figure S6). Therefore, considering both the steric and electronic structure of SDS, we reasoned that a Cu–O interaction would be most likely.^65,66^ With these binding motifs, CTAB would exhibit the weakest interactions with the Cu_2–*x*_Se surface (bond enthalpies: Se–N: 193 kJ, Cu–S: 285 ± 17 kJ, and Cu–O: 343 ± 63 kJ),^67−73^ and exhibit the lowest equilibrium adsorption constant (excluding any additional intermolecular interactions or other surface–ligand interactions). Given this relatively low adsorption constant compared to the other ligands, one would then predict CTAB to exhibit the highest degree of per particle MD surface coverage and the highest percentage of total particles exhibiting modification compared with the other ligands tested. However, while CTAB-terminated Cu_2–*x*_Se NPs do have the highest % surface coverage per NP, they have the lowest % modification of all ligands tested (Figure 3).

This combination of high % surface coverage and low % modification of the CTAB-capped Cu_2–*x*_Se suggests the presence of a cooperative process during MD (vide infra), as opposed to a process mediated by straightforward ligand adsorption equilibria. For example, cooperative dissociation of CTAB from the surface of Cu_2–*x*_Se would explain both that the overall percentage of NPs modified is roughly half that of the other ligands, but that for a particle that *is* modified, its entire surface exhibits Au deposits. Cooperativity of CTAB layers has been observed previously on Au NP surfaces,^74^ where this interaction was attributed to the attractive hydrophobic interactions between the alkyl chains of CTA^+^.

To ensure that our observations concerning the extent of metal deposition were not limited by the amount of available metal ion precursors, we sought to increase the Au:Cu_2–*x*_Se ratio. Here, we hypothesized that if the dissociation of CTAB was cooperative, then increasing the amount of Au precursors in solution should increase the % modification of the colloid, and % surface coverage will also remain high, while for the other 3 ligands, % modification would increase, but % surface coverage would not. Our initial tests using the original synthesis and increasing the Cu_2–*x*_Se:Au ratio did not yield a measurable change in either the percent of particles modified or individual particle surface coverage for any ligand tested. Instead, we observed a marked increase in homogeneously nucleated Au NPs (Figure S9). To mitigate homogeneous Au nucleation but continue to explore the relationship between the available metal and the extent of MD, we performed a series of sequential Au deposition steps on Cu_2–*x*_Se NPs instead of adding all Au precursors at once (described in the Experimental Section above).

**Figure 4:**
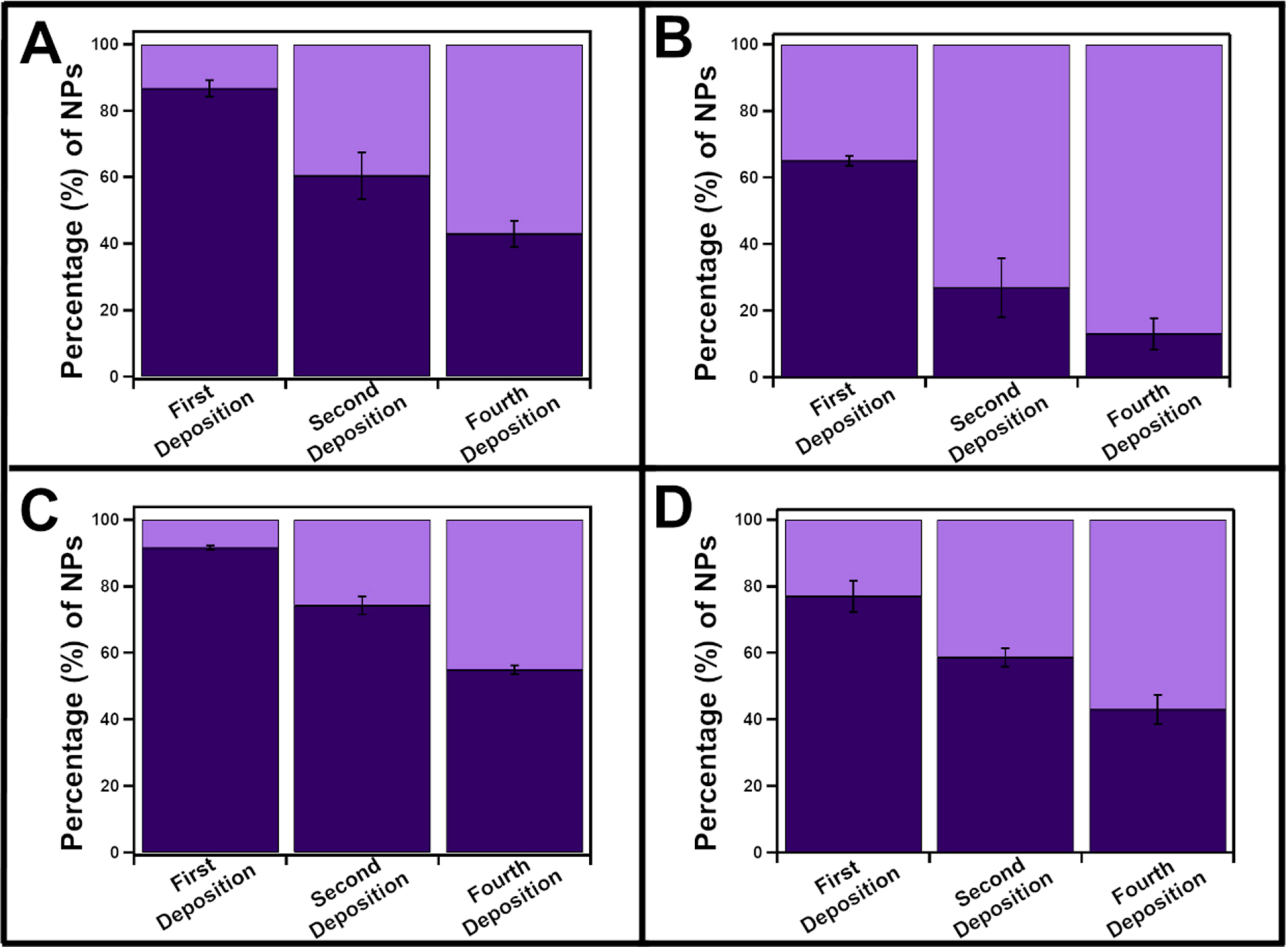
Percent modification of gold metal deposition on Cu_2–*x*_Se NPs capped with (A) CTAB, (B) 3.5 kDa PVP, (C) 1 kDa PEGSH, and (D) SDS for sequential depositions.

**Figure 5:**
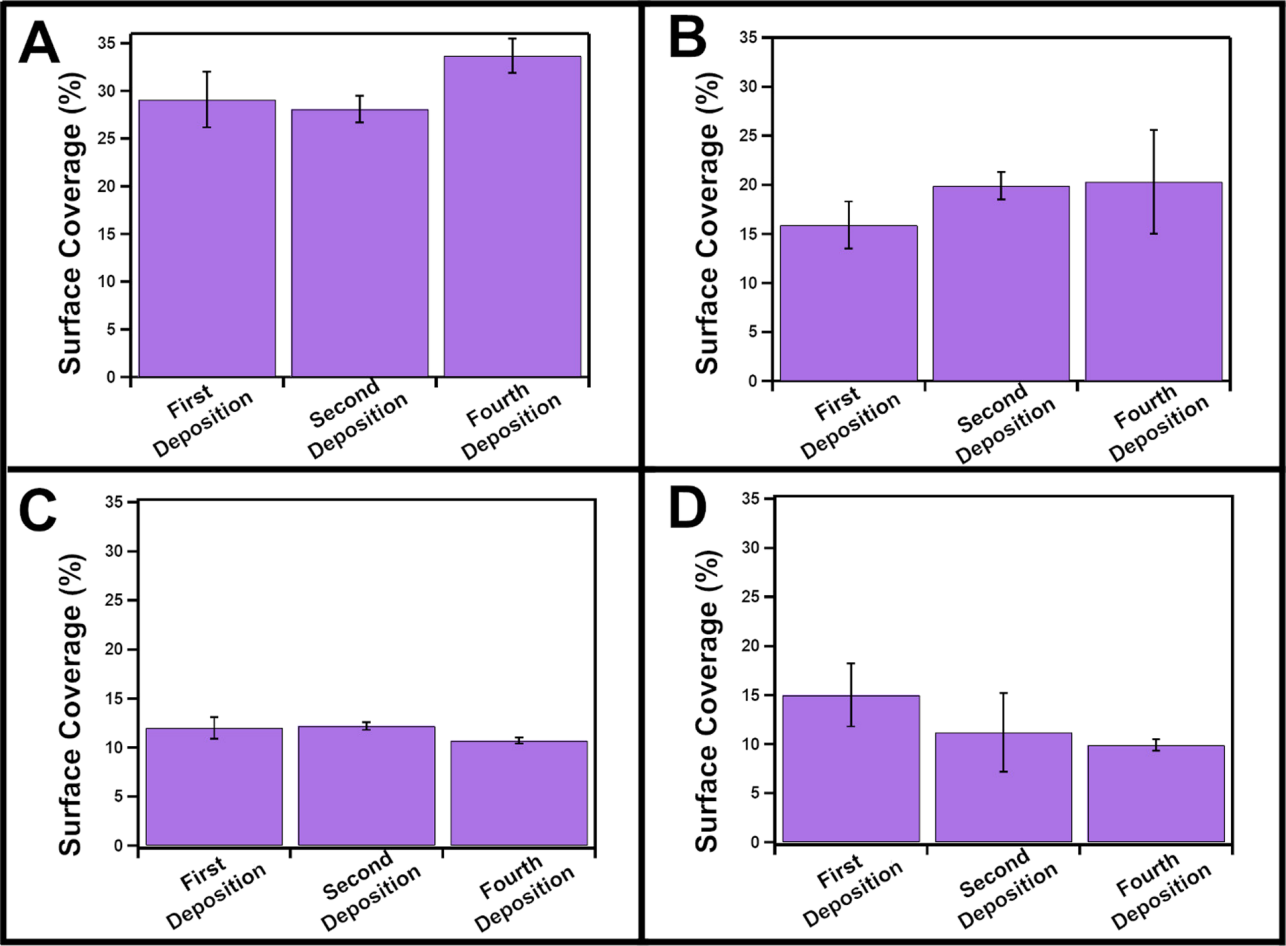
Percent surface coverage of gold metal deposition on Cu_2–*x*_Se NPs capped with (A) CTAB, (B) 3.5 kDa PVP, (C) 1 kDa PEGSH, and (D) SDS for sequential depositions.

Samples from the first, second, and fourth deposition steps were characterized using TEM and statistical analysis to find the % modification and % surface coverage (Figures 4 and 5). Overall, sequential deposition increases the % modification for all ligands, indicating the ability to increase the yield of metal–semiconductor heterostructures in this synthesis.

However, the % surface coverage remains constant over all four deposition steps for all four ligands, and the individual deposits do not exhibit a measurable increase in size (Figure S14). These data indicate that % surface coverage is dependent on the dissociation (and likely dissociation mechanism, such as cooperative dissociation) of the ligand from the surface and independent of the available metal. These results were also consistent for all molecular weights of PVP and PEGSH tested (Figure S23).

Taken together, our results suggest consistent MD outcomes for a variety of Cu_2–*x*_Se surface chemistries in terms of metal island size, shape, number, surface coverage, total particles modified, and response to subsequent deposition steps. Of these surface ligands, only CTAB-terminated Cu_2–*x*_Se NPs exhibited measurably different Au MD morphologies, which we suggest may be due to a cooperative association/disassociation behavior of the CTAB on the Cu_2–*x*_Se surface.

**Figure 6:**
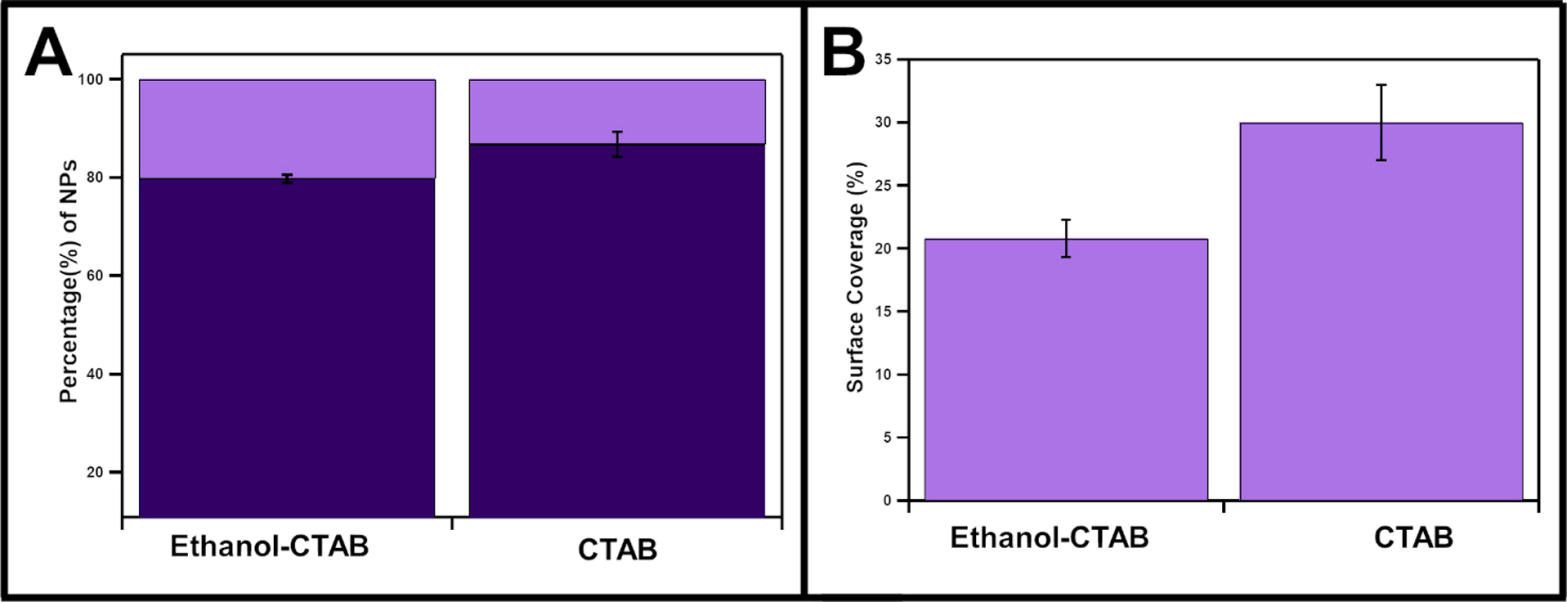
Comparative (A) percent modification and (B) surface coverage of Cu_2–*x*_Se capped with CTAB, with and without the addition of ethanol.

To test this hypothesis, we considered several ways in which we might alter or mimic this cooperativity, including using other alkylammonium halide surfactant ligands and introducing small molecule additives that may disrupt CTAB supramolecular interactions. To test small molecule disruptors, we introduced 50 μL of 200-proof ethanol into 1 mL of CTAB-Cu_2–*x*_Se NP solutions 2 h prior to Au deposition and then proceeded with the Au MD reaction as described above. Under these conditions, we did not observe a change in the metal deposition pattern. However, we did see a slight increase in % modification and a decrease in % surface coverage, which is what would be expected from disrupting the cooperativity of the ligand shell (Figure 6). Although these trends are consistent with disrupting a deposition process dependent on a cooperative surface ligand dissociation mechanism, the effects were too small to change the pattern of MD on CTAB-Cu_2–*x*_Se from isotropic to anisotropic and overarchingly to convince one of a cooperative process. Unfortunately, attempts to increase the concentration of ethanol destabilized the colloid entirely.

In testing additional alkylammonium halide surfactants, we ligand exchanged CTAB-capped Cu_2–*x*_Se NPs with two different cationic alkyl ammonium ligands: trimethyloctylammonium bromide (TMOAB) and tetraoctylammonium bromide (TOAB). These ligands have the same charge and binding group as CTAB, but their carbon chain length and structure are different (straight vs branched). In both cases, we do not expect a full ligand exchange of the CTAB and instead may anticipate varying degrees of disruption to the supramolecular assembly of the CTAB on the NP surface and therefore its cooperative adsorption behavior, similar to the additive experiments above.^75,76^ However, in this case, we may gain some insight via comparison, where one would expect a mixed CTAB-TMOAB ligand shell to show less cooperative dissociation than a pure CTAB shell but more than a CTAB-TOAB shell due to TMOAB containing an *n*-alkane chain that can still participate in intermolecular van der Waals interactions with neighboring CTAB molecules.

**Figure 7:**
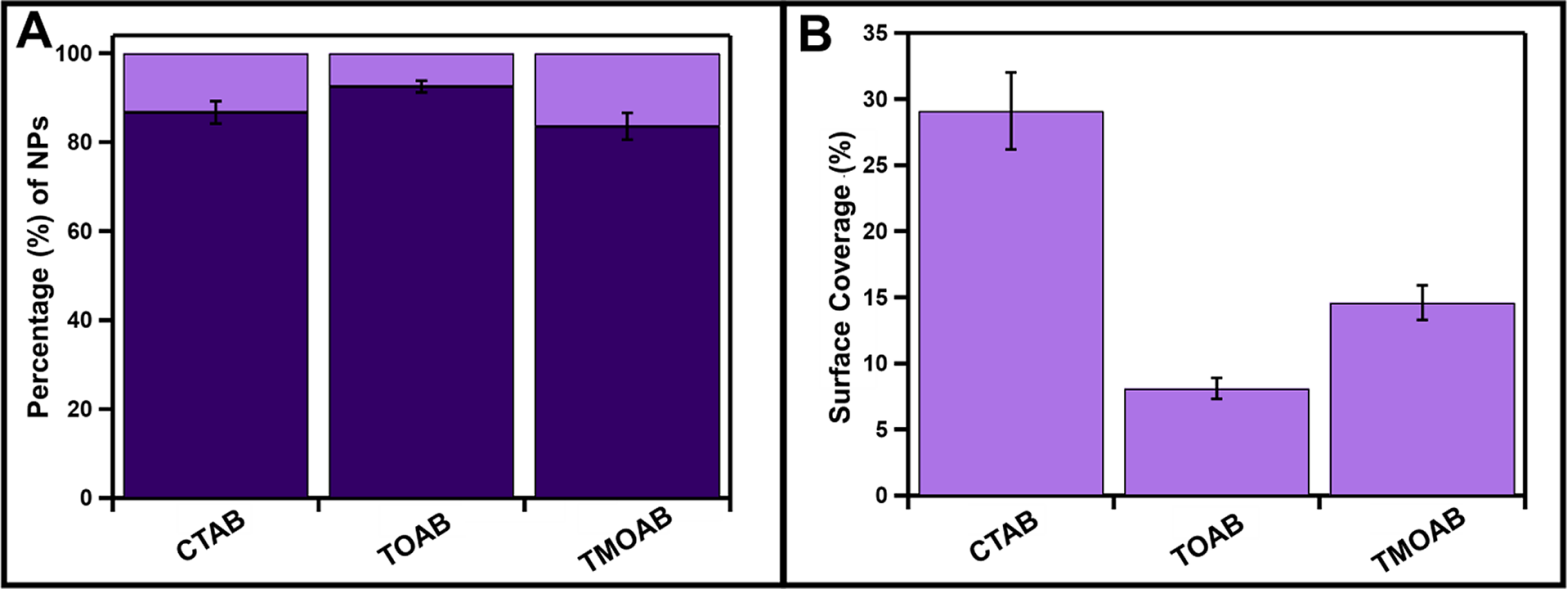
Comparative (A) percent modification and (B) surface coverage of Cu_2–*x*_Se capped with alkylammonium ligands (as-synthesized CTAB and ligand exchanged TOAB and TMOAB).

The % modifications of TMOAB-Cu_2–*x*_Se NPs and TOAB-Cu_2–*x*_Se NPs are within error of CTAB-Cu_2–*x*_Se samples (Figure 7A). Interestingly, the % surface coverage for CTAB-, TMOAB-, and TOAB-Cu_2–*x*_SeNPs decreases in the order one would predict from decreasing cooperative interactions in the ligand layer (CTAB > TMOAB > TOAB) (Figure 7B). These results are also consistent when challenging the system with sequential deposition steps (Figure S32). Overall, these experiments support the role of cooperative CTAB dissociation in the observed MD morphologies.

## Conclusions

Taken together, our results indicate that metal deposition on Cu_2–*x*_Se NPs is surprisingly insensitive to Cu_2–*x*_Se surface chemistry, especially compared to the use of surface chemistry in postsynthetic modification of other NP substrates.^20,28^ We note an outlier in these results: CTAB-modified NPs, which exhibited MD morphologies consistent with a cooperative ligand dissociation process, indicate that cooperative ligand dissociation (and ligand dissociation mechanisms in general) may be a useful (and distinctive) tool in achieving isotropic MD products on Cu_2–*x*_Se NPs.

## Supporting Information Available

Details of NP characterization and statistical analysis methods via UV–vis extinction spectroscopy, TEM, XPS, and size distribution histograms (PDF)
