# Supplementary material for: Influence of Surface Chemistry on Metal Deposition Outcomes in Copper Selenide-Based Nanoheterostructure Synthesis
Source: Langmuir. 2024 Jul 27;40(31):16473–83. doi: 10.1021/acs.langmuir.4c01817 (PMC11308770; doi:10.1021/acs.langmuir.4c01817)
Supplement: Supplementary file 1 — la4c01817_si_001.pdf [file la4c01817_si_001.pdf]

Supporting Information

Influence of surface chemistry on metal deposition outcomes in copper selenide-based nano-heterostructure synthesis

Riti Sen†, Shelby L. Millheim†, Tyler M. Gordon† and Jill E. Millstone†§⊥*

† Department of Chemistry, University of Pittsburgh, Pittsburgh, Pennsylvania 15260, United States.
§ Department of Chemical and Petroleum Engineering, University of Pittsburgh, Pittsburgh, Pennsylvania 15260, United States.
⊥ Department of Mechanical Engineering and Materials Science, University of Pittsburgh, Pittsburgh, Pennsylvania 15260, United States.

Table of Contents:

Characterization of the Ligand Shell 5

Notes on Ligand Concentration Selection 5

Figure S1. (A) Representative UV-vis spectra of the as-synthesized CTAB capped $Cu_{2-x}Se$ NPs and ligand exchanged $Cu_{2-x}Se$ NPs capped with 3.5 kDa PVP, 1 kDa PEGSH and SDS. (B) Representative UV-vis of the as-synthesized CTAB capped $Cu_{2-x}Se$ NPs and ligand exchanged $Cu_{2-x}Se$ NPs capped with 10 kDa PVP, 55 kDa PVP, 5 kDa PEGSH, MUA, MDA, and MBA. 7

Table S1. Representative zeta potential measurements of the as-synthesized CTAB capped $Cu_{2-x}Se$ NPs and ligand exchanged $Cu_{2-x}Se$ NPs capped with 3.5 kDa PVP, 1 kDa PEGSH and SDS. Literature suggests that potentials of less than or equal to |10 mV| should be considered neutral.[12] 7

Figure S2. Powder X-ray diffraction patterns of the as-synthesized (A) CTAB capped $Cu_{2-x}Se$ and ligand exchanged $Cu_{2-x}Se$ NPs capped with (B) 3.5 kDa PVP, (C) 1 kDa PEGSH and (D) SDS. Here the green lines refer to $Cu_{2-x}Se$ (PDF # 00-006-0680). 8

X-ray Photoelectron Spectroscopy (XPS) analysis 8

Figure S3. XPS of the as-synthesized CTAB capped $Cu_{2-x}Se$: (A) N 1s, (B) Se 3d, and (C) Br 3d regions. 9

Figure S4. XPS of the ligand exchanged PEGSH capped $Cu_{2-x}Se$: (A) S 2p, (B) N 1s and (C) Br 3d regions. **9**

Figure S5. XPS of the ligand exchanged PVP-capped $Cu_{2-x}Se$ NPs: (A) O 1s, (B) N 1s, and (C)Se 3d regions. **10**

Figure S6. XPS of the ligand exchanged SDS-capped $Cu_{2-x}Se$: (A) Cu 2p, (B) Se 3d, (C) O 1s, (D) S 2p, (E) Br 3d and (F) N 1s spectra. **10**

Figure S7. Representative TEM images of the (A) as-synthesized CTAB capped $Cu_{2-x}Se$ NPs and subsequent ligand exchanged particles with (B) 3.5kDa PVP (C) 1kDa PEGSH (D) SDS. **11**

Statistical Analysis of Single vs. Multiple Islands on $Cu_{2-x}Se$ NPs 11

Table S2. Statistical analysis of Au island morphology using ANOVA. **12**

Calculating the Percent Surface Coverage 12

Scheme S1. Schematic representation of surface coverage calculation. **12**

Figure S8. Representative TEM images of Pt deposition on $Cu_{2-x}Se$ capped with (A) CTAB (B) 3.5kDa PVP (C) 1kDa PEGSH and (D) SDS. **13**

Figure S9. (A,C) Representative TEM image of homogeneous Au and Pt nucleation on increase Au/Pt to $Cu_{2-x}Se$ ratio; (B,D) % modification and % surface coverage of $Cu_{2-x}Se$ NPs. **14**

Figure S10. Representative TEM images of the $Cu_{2-x}Se$ (A) and first (B), second (C) and fourth (D) sequential gold deposition on $Cu_{2-x}Se$ NPs capped with CTAB. **15**

Figure S11. Representative TEM images of the $Cu_{2-x}Se$ (A) and first (B), second (C) and fourth (D) sequential gold deposition on $Cu_{2-x}Se$ NPs capped with 3.5kDa PVP. **16**

Figure S12. Representative TEM images of the $Cu_{2-x}Se$ (A) and first (B), second (C) and fourth (D) sequential gold deposition on $Cu_{2-x}Se$ NPs capped with 1kDa PEGSH. **17**

Figure S13. Representative TEM images of the $Cu_{2-x}Se$ (A) and first (B), second (C) and fourth (D) sequential gold deposition on $Cu_{2-x}Se$ NPs capped with SDS. **18**

Size Distributions of Particles and Deposition Islands 18

Table S3. Size distribution of core nanoparticle (diameter) and length of deposition island (length) (first deposition step). **18**

Fig S14. Histograms of size distributions of gold islandic deposition length from sequential deposition on $Cu_{2-x}Se$ capped with (A,B,C) CTAB (D,E,F) 3.5kDa PVP (G,H,I) 1kDa PEGSH and (J,K,L) SDS. **20**

Figure S15. Histograms of size distributions of (A) as-synthesized CTAB capped $Cu_{2-x}Se$ and ligand exchanged $Cu_{2-x}Se$ with (B) 3.5kDa PVP, (C) 1kDa PEGSH and (D) SDS. **21**

Figure S16. Histograms of size distributions of ligand exchanged $Cu_{2-x}Se$ and subsequent Au deposition length on $Cu_{2-x}Se$ capped with (A,D) 10kDa PVP (B,E) 55kDa PVP, and (C,F) 5kDa PEGSH. 21

Figure S17. Histograms of size distributions of ligand exchanged $Cu_{2-x}Se$ and subsequent gold islandic deposition length on $Cu_{2-x}Se$ capped with (A) MUA (B) MDA, and (C) MBA. 22

Figure S18. Histograms of size distributions and subsequent Au deposition length on $Cu_{2-x}Se$ capped with (A,D) TOAB (ligand exchanged) (B,E) TMOAB (ligand exchanged), and (C,F) TMOAB (as-synthesized). 22

Figure S19. Histograms of size distributions of gold islandic deposition length from sequential deposition on $Cu_{2-x}Se$ capped with (A,B,C) 10kDa PVP (D,E,F) 55kDa PVP and (G,H,I) 5kDa PEGSH. 23

Figure S20. Histograms of size distributions of gold islandic deposition length from sequential deposition on $Cu_{2-x}Se$ capped with (A,B,C) MUA (D,E,F) MDA and (G,H,I) MBA. 23

Figure S21. Histograms of size distributions of gold islandic deposition length from sequential deposition on $Cu_{2-x}Se$ capped with (A,B) TOAB (ligand exchanged) (C,D) TMOAB (ligand exchanged) and (E,F) TMOAB (as-synthesized). 24

Figure S22. Percent surface coverage and percent modification of gold metal deposition on $Cu_{2-x}Se$ NPs capped with (A,B) PVP and (C,D) PEGSH. 25

Figure S23. Percent modification and surface coverage of sequential gold metal deposition on $Cu_{2-x}Se$ NPs capped with (A,D) 55kDa PVP, (B,E) 10kDa PVP, and (C,F) 5kDa PEGSH for sequential depositions. 25

Figure S24. Representative TEM images of the $Cu_{2-x}Se$ (A) and first (B), second (C) and fourth (D) sequential gold deposition on $Cu_{2-x}Se$ NPs capped with 10kDa PVP. 26

Figure S25. Representative TEM images of the $Cu_{2-x}Se$ (A) and first (B), second (C) and fourth (D) sequential gold deposition on $Cu_{2-x}Se$ NPs capped with 55kDa PVP. 27

Figure S27. Representative TEM images of the $Cu_{2-x}Se$ (A) and first (B), and second (C) sequential gold deposition on $Cu_{2-x}Se$ NPs ligand exchanged with TMOAB. 29

Figure S28. Representative TEM images of the $Cu_{2-x}Se$ (A) and first (B), and second (C) sequential gold deposition on $Cu_{2-x}Se$ NPs ligand exchanged with TOAB. 29

Figure S29. Representative TEM images of the $Cu_{2-x}Se$ (A) and first (B), second (C) and fourth (D) sequential gold deposition on $Cu_{2-x}Se$ NPs capped with MBA. 30

Figure S30. Representative TEM images of the $Cu_{2-x}Se$ (A) and first (B), second (C) and fourth (D) sequential gold deposition on $Cu_{2-x}Se$ NPs capped with MDA. 31

Figure S31. Representative TEM images of the $Cu_{2-x}Se$ (A) and first (B), second (C) and fourth (D) sequential gold deposition on $Cu_{2-x}Se$ NPs capped with MUA. **32**

Figure S32. Comparative (A) percent modification and (B) surface coverage of $Cu_{2-x}Se$ functionalized with TMOAB (ligand exchanged from CTAB). **33**

References **33**

Characterization of the Ligand Shell

In this report, we investigate the effects of a ligand shell on the post-synthetic deposition morphology of Au and Pt on colloidal $Cu_{2-x}Se$ NPs. Of course, to make the most robust structure-synthesis correlations, we aimed to characterize the ligand shell with the detail necessary to make those correlations. In particular, we sought to determine the zeta-potential, thickness, and chemical composition of each ligand shell tested. Below, we elaborate on challenges encountered while conducting these analyses as a footnote to our discussions in the main text.

To determine the density and identity of small molecule ligands on the NP surface quantitatively, our preferred method uses a 1D, solution phase ^{1}H-NMR based approach which has the advantage of not only giving the number of ligands per particle, but also being able to distinguish the variety of ligands present in most cases.[1] Unfortunately, the Cu^{2+} content of $Cu_{2-x}Se$ NPs makes this analysis unfeasible due to peak broadening from the paramagnetic cation.[2]

Thermogravimetric analysis (TGA) is another possible technique to quantify the ligand shell for metallic nanoparticles, at least in terms of mass. However, this method is limited in the characterization of multicomponent ligand shells (*i.e.* particle ligand shells that contain more than one ligand type), since often it is not possible to distinguish between ligand removal temperatures. We attempted to use TGA to analyze the ligand shell compositions but found high variation from sample to sample. The method also required large amounts of material for analysis (> 5-10 mg of conjugated $Cu_{2-x}Se$ NPs per experiment), and this barrier combined with the limited insight of the results, led us to explore alternative methods.

Zeta potential measurements support ligand exchange from CTAB to other ligands, where measured changes in zeta potential are consistent with each ligand charge (**Table S1**). These results are further supported by X-ray photoelectron spectroscopy (XPS) analysis. XPS was useful in determining both what was and was not present on the $Cu_{2-x}Se$ NP surfaces. In particular, it was important to note that we did not observe evidence of surface-bound bromide (or chloride or iodide) on any of the NP surfaces including those NPs functionalized with CTAB. Nitrogen signals indicating surface bound quaternary ammonium species observed in the CTAB-functionalized $Cu_{2-x}Se$ NP samples were not present after ligand exchange with either PEGSH, PVP, or SDS (**Figure S3-6**). XPS also indicates the presence of S-Cu(II) interactions in the case of PEGSH, and O-Cu(II) interactions in the case of PVP.

Taken together, the data support ligand exchange from CTAB to other ligand chemistries, with low or no levels of detectable CTAB after exchange. However, a quantitative picture of the ligand shell chemistries could not be obtained within the scope of a single report.

Notes on Ligand Concentration Selection

Ligand concentrations used for the ligand exchange were guided by three factors: 1) that ligand exchange occurs via mass action ligand exchange, and therefore, 2) the concentration should be in vast excess of theoretically available nanoparticle surface

area as determined by particle concentration and theoretically determined minimum ligand footprint; as shown by several works concerning ligand functionalization on many types of nanoparticle surfaces,[1, 3-10] and 3) the concentration of ligand needed to obtain the most reproducible particle properties (e.g. XPS spectra, zeta potential, and extinction spectra) across multiple, independent trials.

In our experiments, the calculation of theoretical footprint is straightforward for the small molecule ligands tested and we have reported this approach in previous work.[1, 11] However, the calculation of theoretical maximum ligand loading in the case of polymeric ligands is, by definition, more complex because multiple ligand-surface interactions are possible per polymer chain. Therefore, we benchmarked our ligand excess to 100 times available surface area for our smallest footprint ligand.

In addition to these considerations, we took into consideration the varying affinities of the ligands for the NP surface, which impact the efficiency of the mass action ligand exchange. For example, a ligand with a weaker binding energy to the $Cu_{2-x}Se$ surface will need a larger ligand excess to achieve the same extent of ligand exchange when compared to a high binding affinity ligand. Therefore, ligands such as SDS required a larger excess compared to thiol-containing ligands.

For polymeric ligands, one can assess concentration comparison to the small molecule ligands either by benchmarking their concentration with respect to polymer chain concentration or to monomer unit concentration. Using either metric, there is some deviation from direct comparison to the ligand exchange conditions for small molecule ligands.

Taken together, these factors represent a wide set of conditions that make using the same concentration of each ligand during ligand exchange a factor that is likely to introduce more variation than it prevents. Instead, the concentrations listed in our experimental section represent a vast excess of ligand with respect to surface area in all cases, and then are additionally modified to concentrations empirically determined to give both the most reproducible particle properties (e.g. zeta potential, X-ray photoelectron spectra, and extinction spectra) and final NP morphologies.

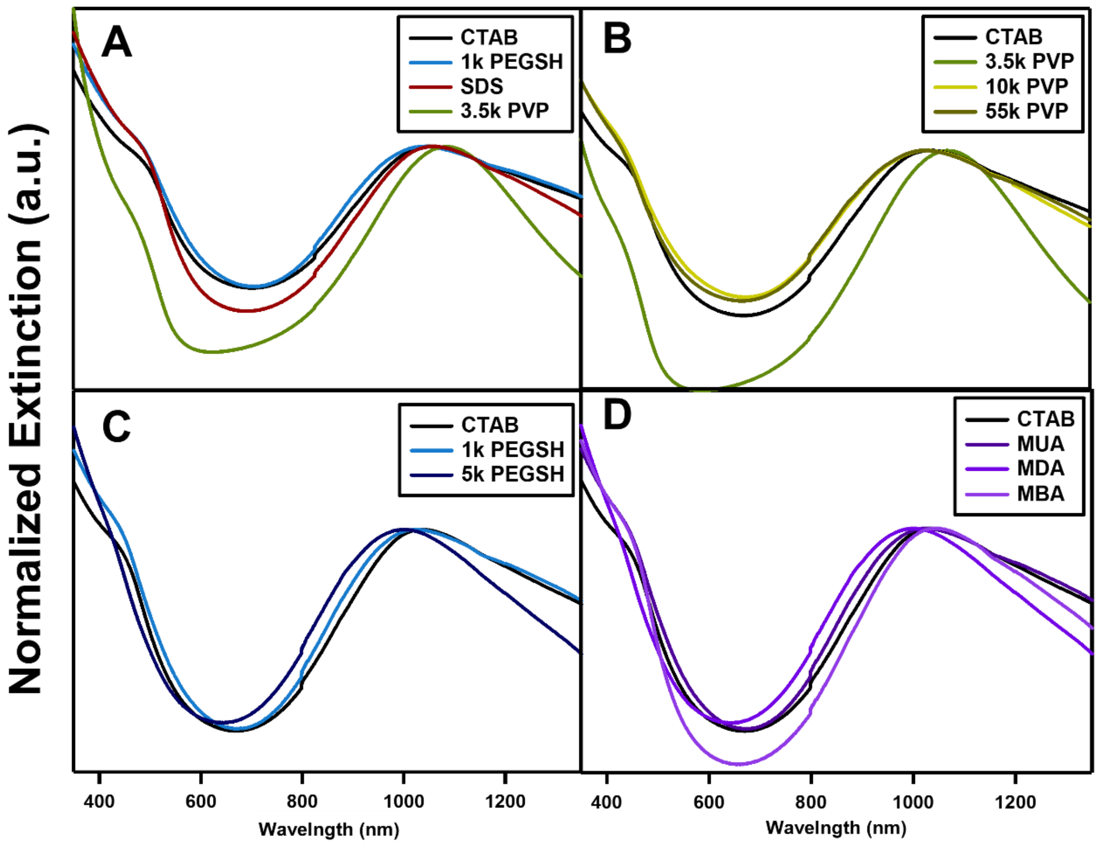

Figure S1. (A) Representative UV-vis spectra of the as-synthesized CTAB capped $Cu_{2-x}Se$ NPs and ligand exchanged $Cu_{2-x}Se$ NPs capped with 3.5 kDa PVP, 1 kDa PEGSH and SDS. (B) Representative UV-vis of the as-synthesized CTAB capped $Cu_{2-x}Se$ NPs and ligand exchanged $Cu_{2-x}Se$ NPs capped with 10 kDa PVP, 55 kDa PVP, 5 kDa PEGSH, MUA, MDA, and MBA.

Table S1. Representative zeta potential measurements of the as-synthesized CTAB capped $Cu_{2-x}Se$ NPs and ligand exchanged $Cu_{2-x}Se$ NPs capped with 3.5 kDa PVP, 1 kDa PEGSH and SDS. Literature suggests that potentials of less than or equal to |10 mV| should be considered neutral.[12]

Ligand	Charge (mV)
CTAB	102.0 ± 0.7
3.5k PVP	2.1 ± 0.6

**10k PVP**	**1.5 ± 0.6**
**1k PEGSH**	**-7.0 ± 1.0**
**5k PEGSH**	**-7.1 ± 0.5**
**SDS**	**-36.5 ± 1.3**

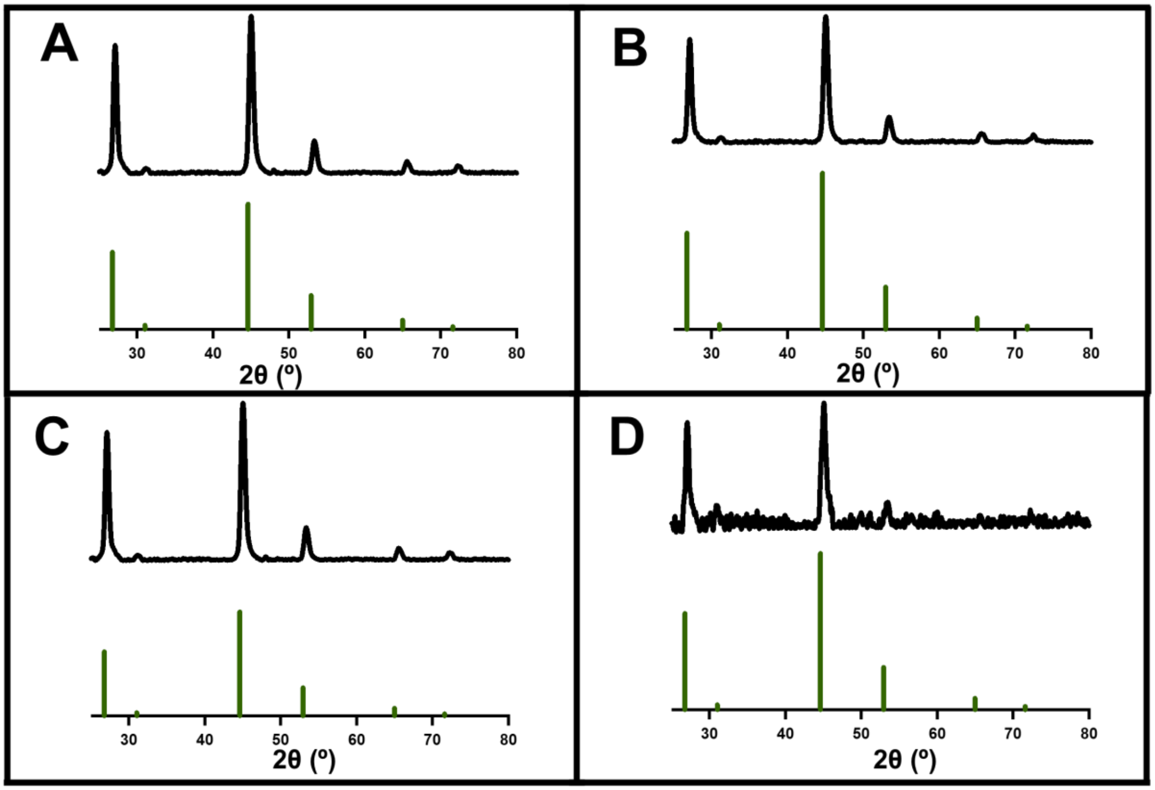

Figure S2. Powder X-ray diffraction patterns of the as-synthesized (A) CTAB capped $Cu_{2-x}Se$ and ligand exchanged $Cu_{2-x}Se$ NPs capped with (B) 3.5 kDa PVP, (C) 1 kDa PEGSH and (D) SDS**.** Here the green lines refer to $Cu_{2-x}Se$ (PDF # 00-006-0680).

X-ray Photoelectron Spectroscopy (XPS) analysis

XPS spectra of CTAB-$Cu_{2-x}Se$ NPs (**Figure S3**), shows no presence of halide on the surface of the $Cu_{2-x}Se$ NPs. The N 1s spectra shows two peaks at 399.3 eV and 402.7 eV. The peak at ~399 eV is attributed to the Se-N interaction that arises from the CTA^+ with the Se^{4-} at the surface of the NPs.[13-15] The Se 3d spectra of bare $Cu_{2-x}Se$ materials typically exhibits Se^{4-} peaks at ~53.8 eV.[16] For our CTAB-capped $Cu_{2-x}Se$, the Se $3d_{5/2}$

is shifted to slightly higher binding energy (~54.2 eV), which corresponds to the Se-N interaction from the CTA^+ moiety.

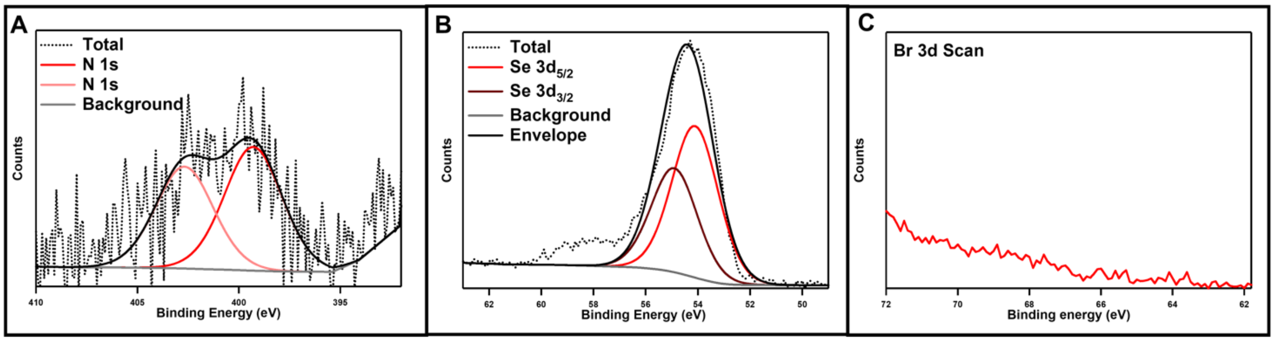

Figure S3. XPS of the as-synthesized CTAB capped $Cu_{2-x}Se$: (A) N 1s, (B) Se 3d, and (C) Br 3d regions.

For the XPS spectra of PEGSH-$Cu_{2-x}Se$ NPs, S $2p_{3/2}$ spectra show two distinct peaks at 162 eV and 166.4 eV (**Figure S4**). The peak at 162 eV corresponds to a metal-thiol interaction that indicates that PEGSH binds through the S atom.[17] The higher binding energy peak at 166.4 eV corresponds to the presence of oxidized thiol species. N and Br regions did not show resolvable peaks suggesting a thorough ligand exchange to PEGSH.

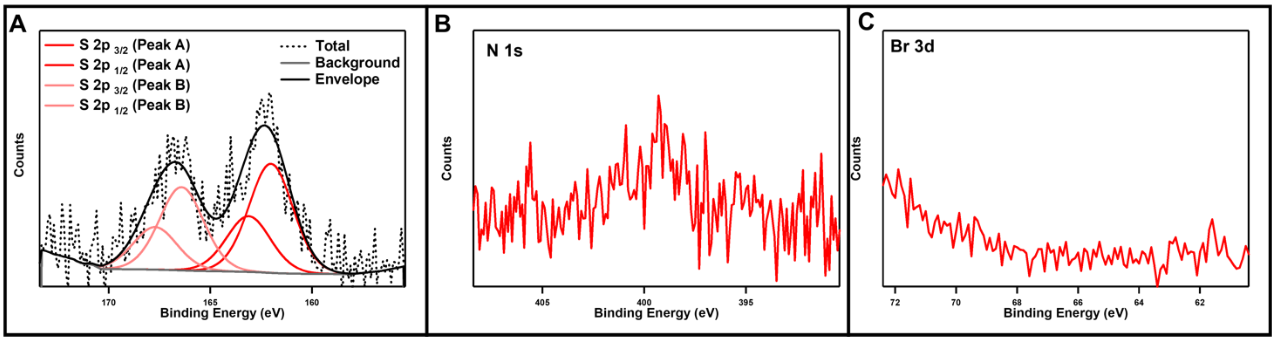

Figure S4. XPS of the ligand exchanged PEGSH capped $Cu_{2-x}Se$: (A) S 2p, (B) N 1s and (C) Br 3d regions.

From the XPS spectra of PVP-$Cu_{2-x}Se$ we can conclude that the interaction with $Cu_{2-x}Se$ surface likely occurs through the oxygen and not through the nitrogen in the pyrrolidone repeat unit (**Figure S5**). The O 1s spectrum shows two distinct binding energies at 530.9 eV and 532.6 eV. Literature reports suggest that unbound carbonyl in the pyrrolidone of PVP exhibits binding energies in the 530-531 eV range.[18, 19] If the carbonyl O interacts with the Cu surface one would expect a shift to higher binding energies, and we observe a shift at higher binding energy (532.6 eV) consistent with that interaction. The N 1s spectrum also shows only one species, with a chemical shift that matches references to free PVP(C-N) as opposed to residual CTAB.[19] Se 3d peaks for PVP-$Cu_{2-x}Se$ only show one binding environment at 54 eV, which suggests that

there is only one type of Se^{4-} environment and that there is no CTAB-Se^{4-} interaction (*N.B.* The peak at ~59 eV corresponds to an oxidized Se^{2+} species which likely arises from degraded NPs). Taken together, these XPS results are consistent with thorough ligand exchange of CTAB to PVP.

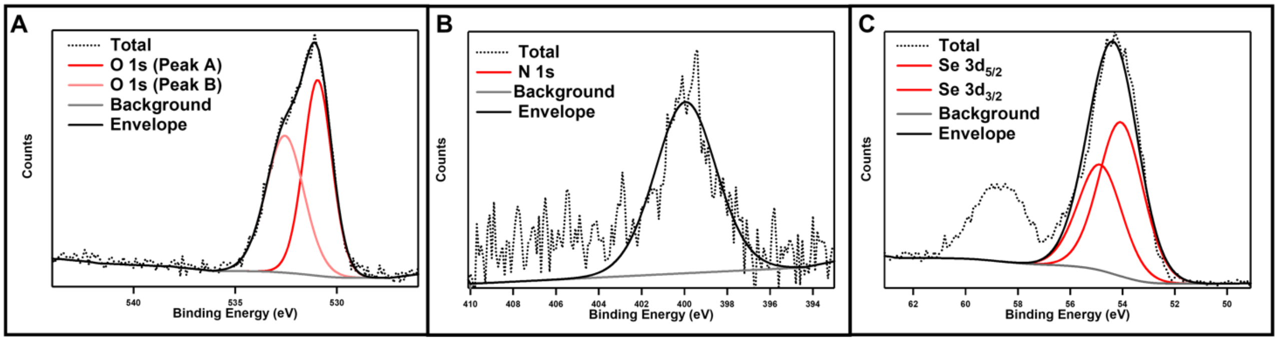

Figure S5. XPS of the ligand exchanged PVP-capped $Cu_{2-x}Se$ NPs: (A) O 1s, (B) N 1s, and (C)Se 3d regions.

The N 1s and Br 3d spectra of the SDS-capped $Cu_{2-x}Se$ NPs show that there is no evidence of CTAB on the surface indicating a complete exchange (**Figure S6**). The Cu 2p spectra shows the presence of both Cu(I) and Cu(II) species, but the exact binding moiety for SDS is not clear. The S 2p spectrum contains peaks that correspond to free SDS (Peak A at ~168.4 eV)[20] and some reduced sulfur species (peaks B and C). If SDS was binding through the S group to the $Cu_{2-x}Se$ surface, we would expect a S species at a higher binding energy than free SDS (>169 eV). Since we do not observe any higher binding energy species, we hypothesize that SDS binds through the O group to the surface of $Cu_{2-x}Se$. The O1s spectrum shows only one species which is within the range of SDS's sulfate group but we cannot definitively assign this interaction.[20, 21]

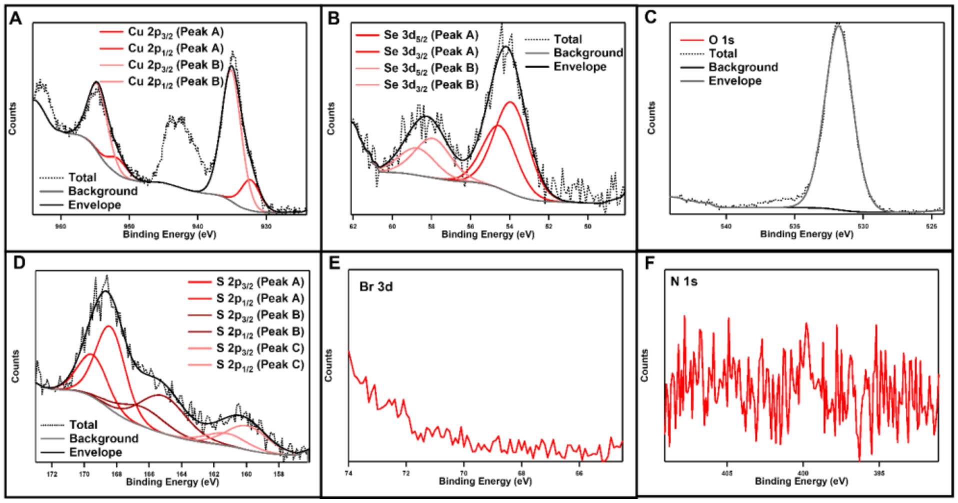

Figure S6. XPS of the ligand exchanged SDS-capped $Cu_{2-x}Se$: (A) Cu 2p, (B) Se 3d, (C) O 1s, (D) S 2p, (E) Br 3d and (F) N 1s spectra.

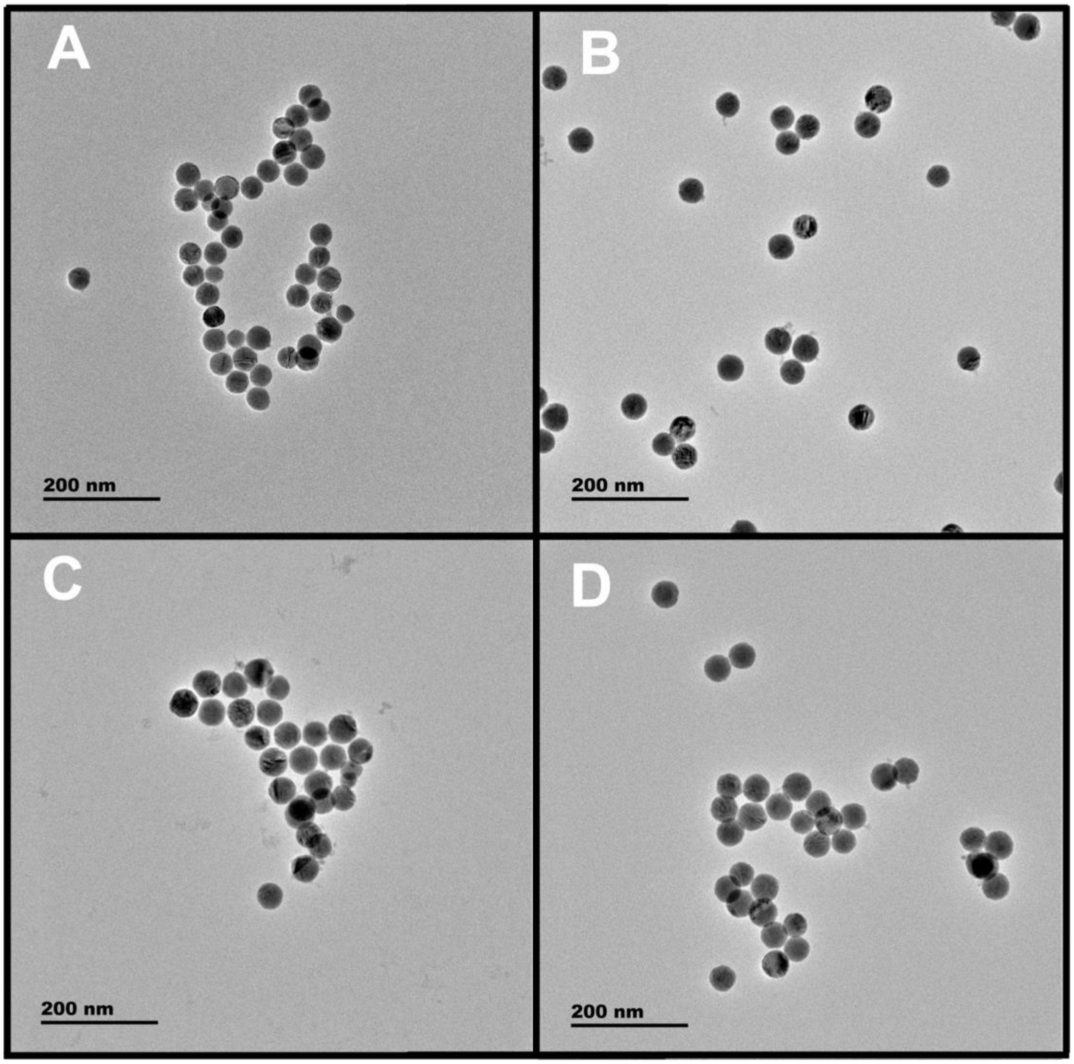

Figure S7. Representative TEM images of the (A) as-synthesized CTAB capped $Cu_{2-x}Se$ NPs and subsequent ligand exchanged particles with (B) 3.5kDa PVP (C) 1kDa PEGSH (D) SDS.

Statistical Analysis of Single vs. Multiple Islands on $Cu_{2-x}Se$ NPs

Single factor analysis of variance (ANOVA) was performed using Microsoft Excel 2017 Analysis ToolPak. This method is used to assess whether multiple populations have the same mean by comparing the variation between the means of multiple samples to the variation within each sample.[22] The null hypothesis for this experiment is that measured Au island distributions are statistically the same. Here, we use two figures of merit to determine if our sample populations are statistically the same. First, if the p value is larger than a given confidence level, a (in this case, 95%, a = 0.05), then the null hypothesis cannot be rejected. Next, if the value (denoted F) produced by this

sample is below a critical value (denoted $F_{critical}$) for a given confidence level (a = 0.05), the means of each population are statistically the same.

The ANOVA derived parameters for Au depositions on PVP, PEGSH and SDS capped NP are statistically the same with one another. However, CTAB is statistically distinct from the other three ligands.

Table S2. Statistical analysis of Au island morphology using ANOVA.

	All Ligands	3.5k PVP, 1k PEGSH and SDS
***p* value**	0.0001	0.5588
**F**	4.0661	0.4055
**$F_{critical}$**	39.39	7.7086
**RESULTS**	**STATISTICALLY DIFFERENT**	**STATISTICALLY SAME**

Calculating the Percent Surface Coverage

We have calculated the average percentage surface area of an individual $Cu_{2-x}Se$ NP that is covered by Au islands. To calculate this value, we estimated each Au island as a hemisphere as an approximation of the actual pseudospherical morphologies observed by TEM in each deposition case. (**Scheme S1**)

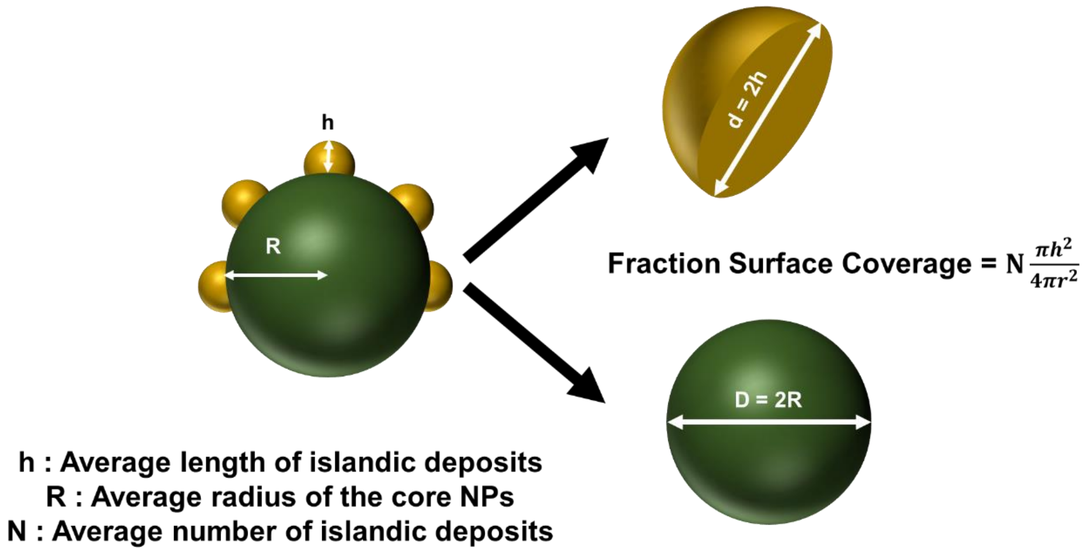

Scheme S1. Schematic representation of surface coverage calculation.

We assume that the length (h) of the Au island is the radius of the hemisphere, which is measured from the TEM images using ImageJ software. Using the area of a circle and

this value as the radius, we get the average surface area of a $Cu_{2-x}Se$ NP covered by each Au island. The next parameter we measure from the TEM images is the average number of Au deposits on each $Cu_{2-x}Se$ NP (N). The total surface area of the $Cu_{2-x}Se$ NPs covered by the Au islandic deposits is given by: $N\pi h^2$.

To better compare this value between different surface chemistries of $Cu_{2-x}Se$, we express surface coverage as a percentage of the total surface area of a $Cu_{2-x}Se$ NP. The average total surface area of a $Cu_{2-x}Se$ NP is calculated from the TEM size distributions as: $4\pi R^2$, where R is the average radius of the NP core. Putting these values together, the percentage of surface coverage is given by: $(\frac{N\pi h^2}{4\pi R^2} \times 100)$

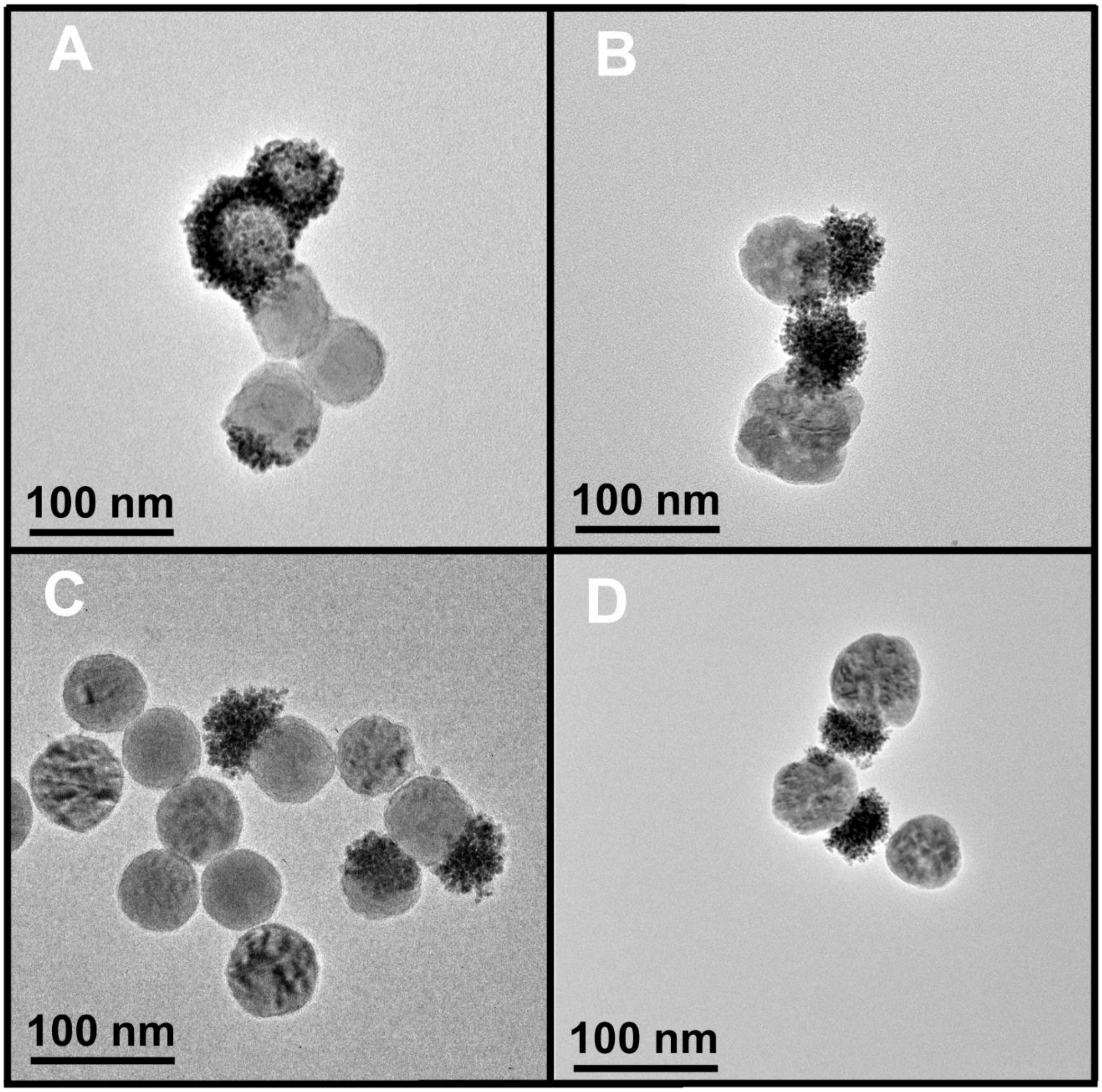

Figure S8. Representative TEM images of Pt deposition on $Cu_{2-x}Se$ capped with (A) CTAB (B) 3.5kDa PVP (C) 1kDa PEGSH, and (D) SDS.

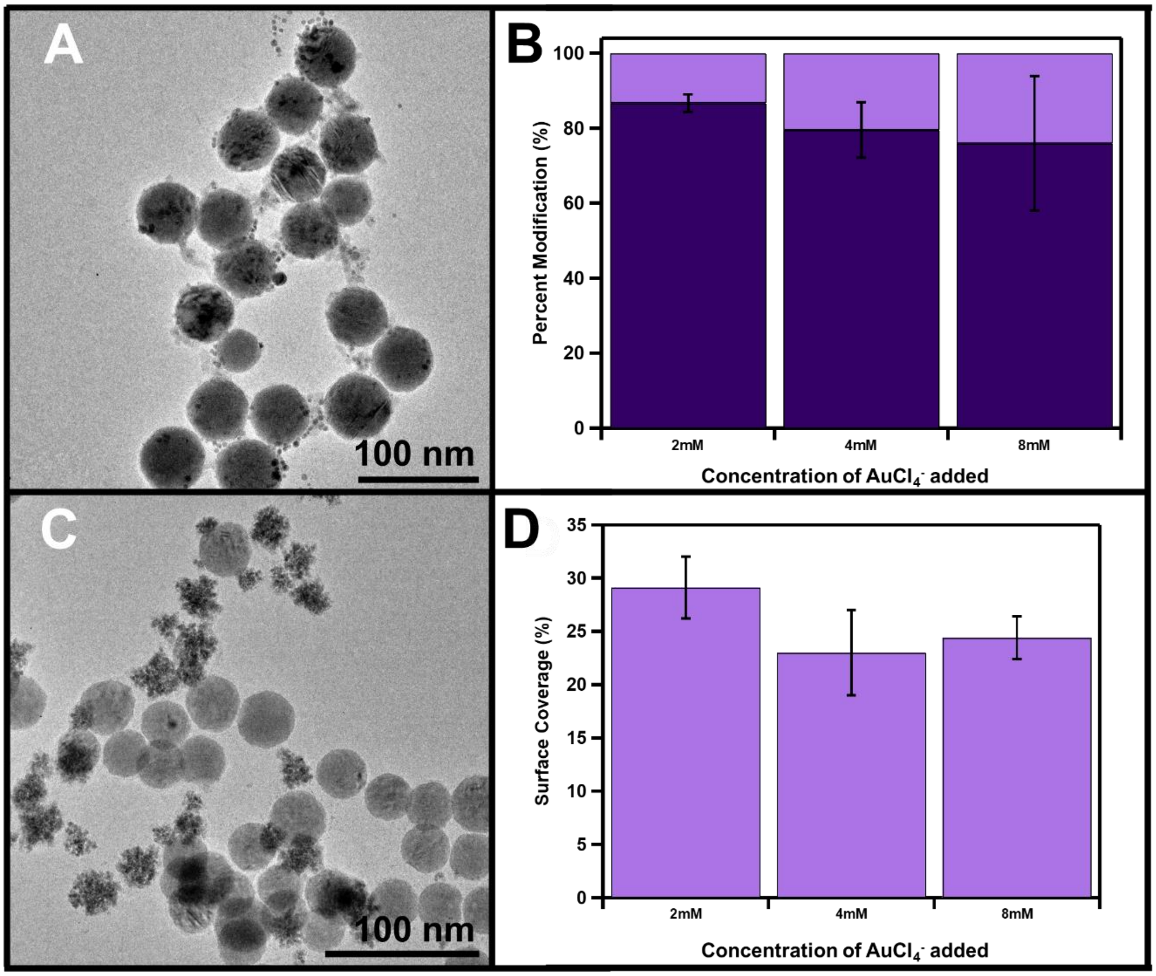

Figure S9. (A,C) Representative TEM images of homogeneous Au and Pt nucleation on increasing the Au/Pt to $Cu_{2-x}Se$ ratio; (B,D) % modification and % surface coverage of $Cu_{2-x}Se$ NPs.

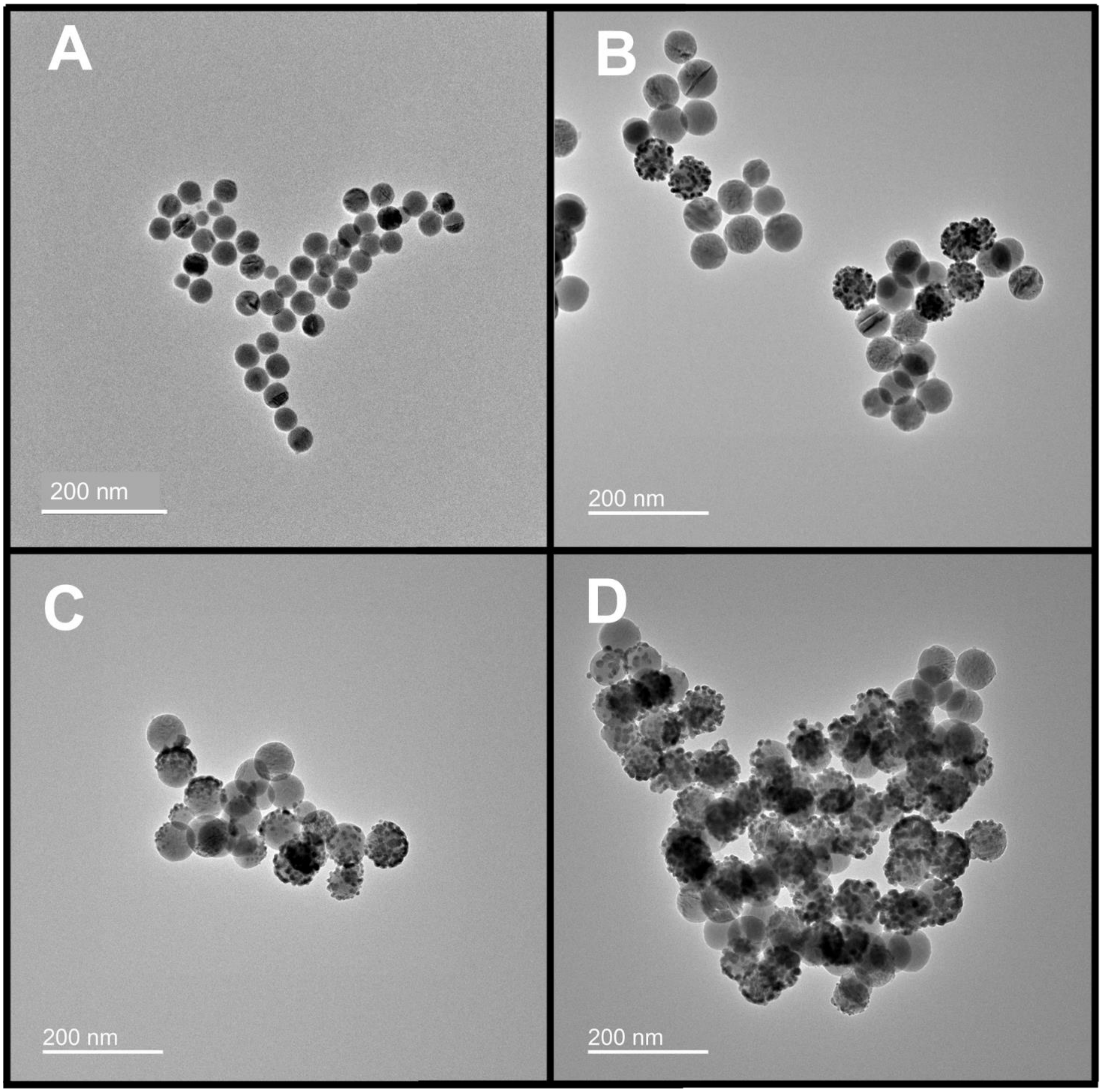

Figure S10. Representative TEM images of the $Cu_{2-x}Se$ (A) and first (B), second (C) and fourth (D) sequential gold deposition on $Cu_{2-x}Se$ NPs capped with CTAB.

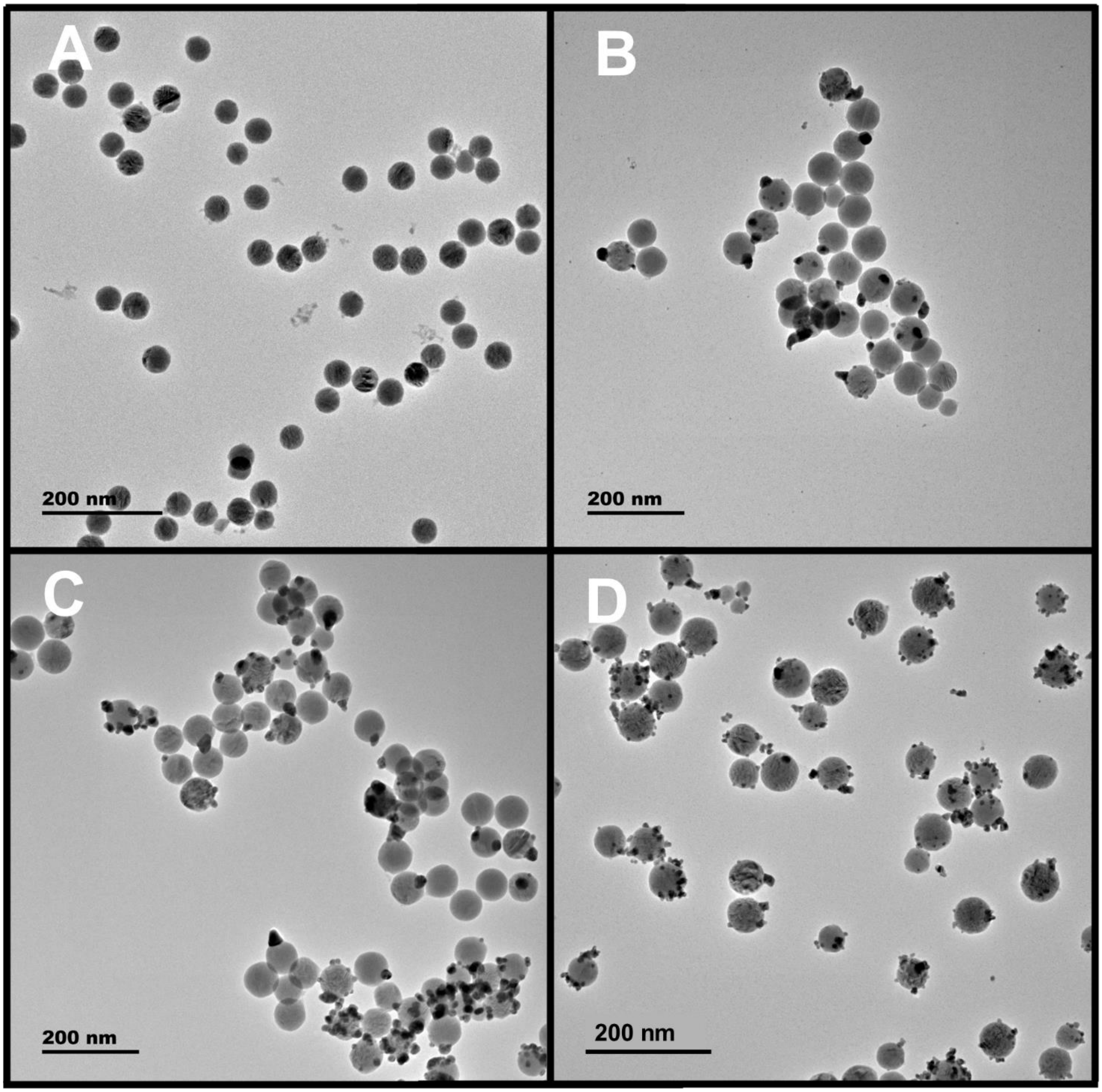

Figure S11. Representative TEM images of the $Cu_{2-x}Se$ (A) and first (B), second (C), and fourth (D) sequential gold deposition on $Cu_{2-x}Se$ NPs capped with 3.5kDa PVP.

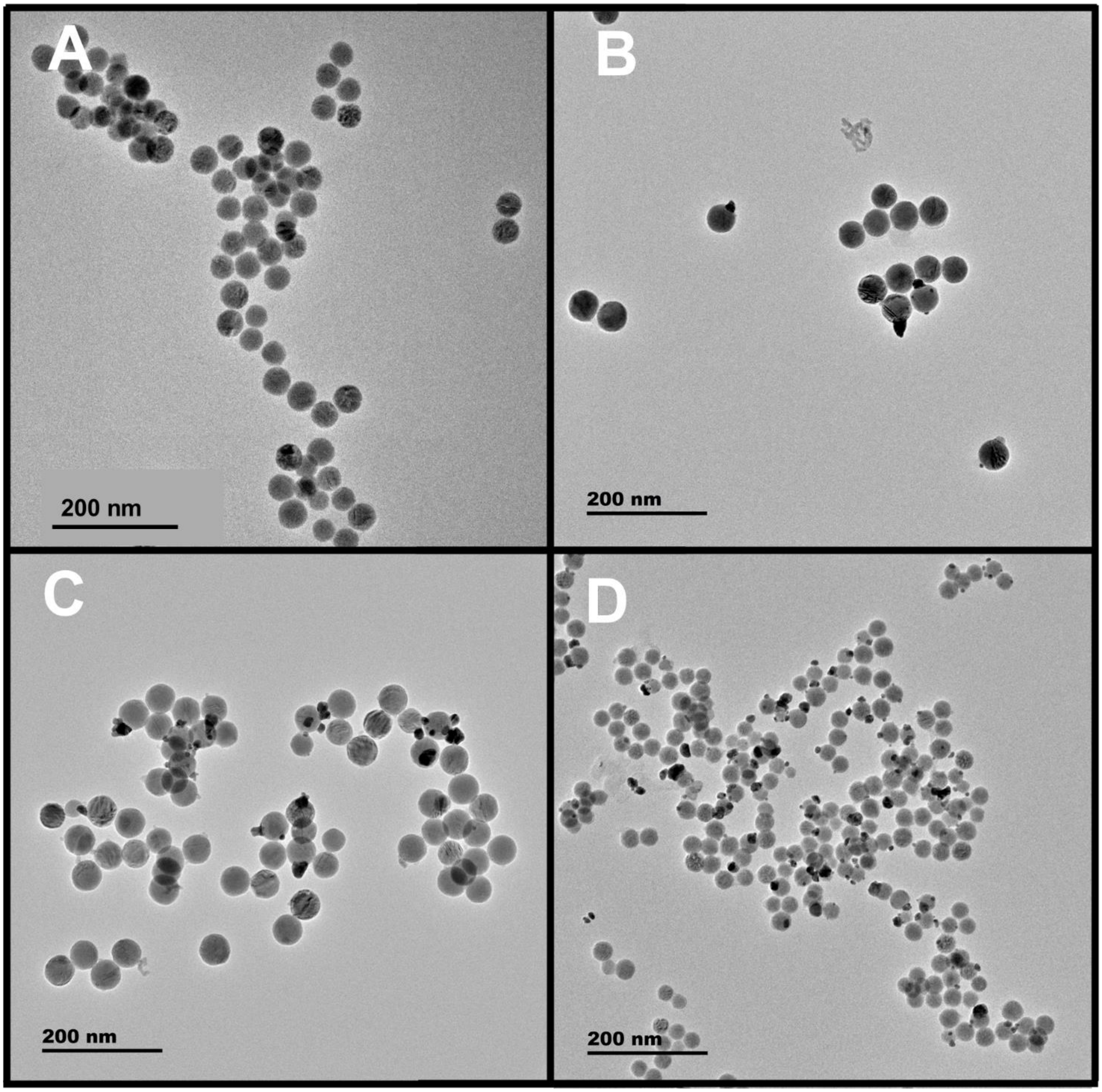

Figure S12. Representative TEM images of the $Cu_{2-x}Se$ (A) and first (B), second (C) and fourth (D) sequential gold deposition on $Cu_{2-x}Se$ NPs capped with 1kDa PEGSH.

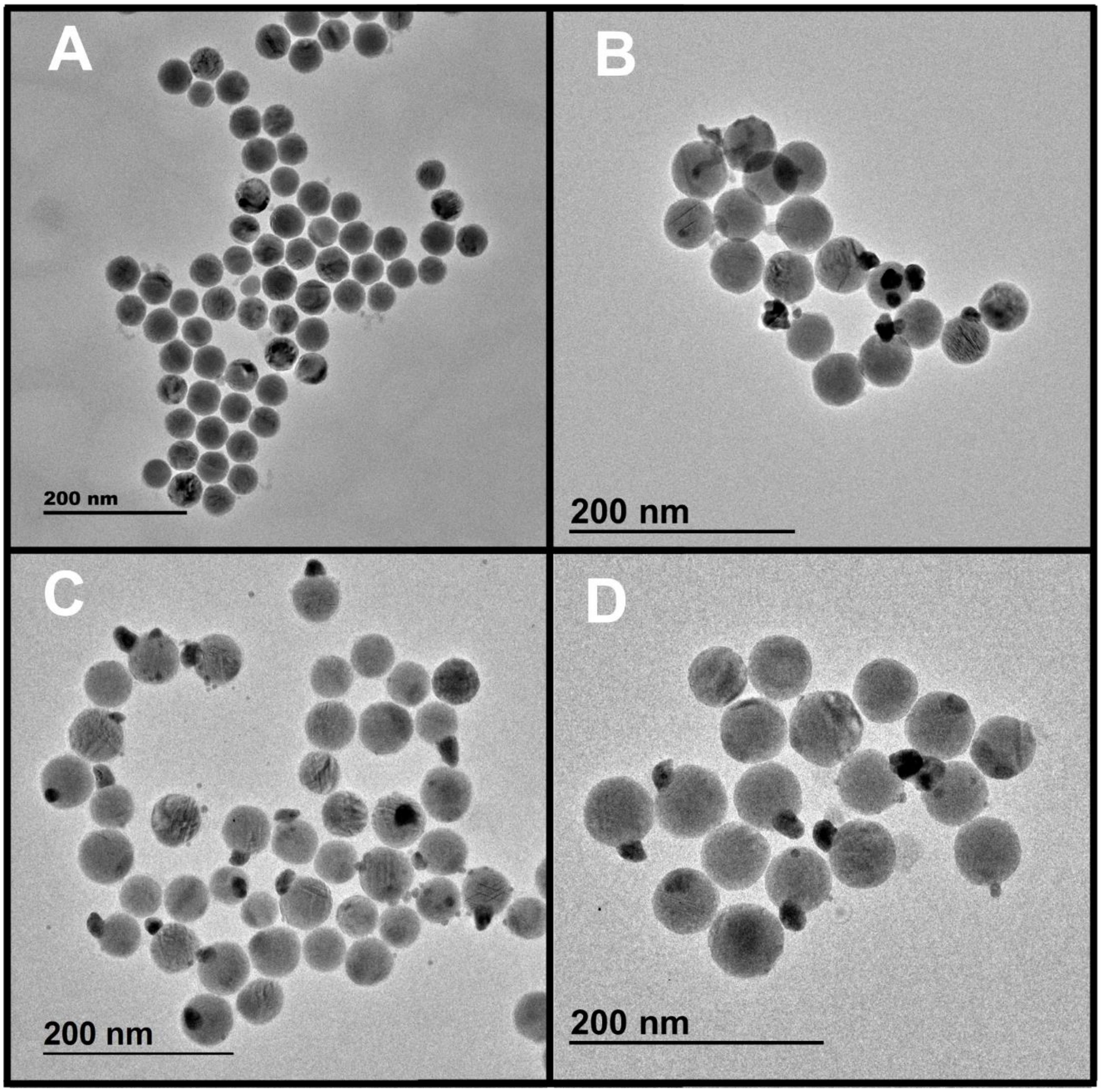

Figure S13. Representative TEM images of the $Cu_{2-x}Se$ (A) and first (B), second (C), and fourth (D) sequential gold deposition on $Cu_{2-x}Se$ NPs capped with SDS.

Size Distributions of Particles and Deposition Islands

The size distributions of the core particle and metallic island deposits were analyzed using over 200 particles for each sample. Sizes are represented below both in histogram and table form for reader convenience.

Table S3. Size distributions of core nanoparticles (diameter) and deposition islands (length) (first deposition step).

Ligands	Diameter (nm)	Length (nm)
CTAB	45 ± 4	10 ± 3
3.5kDa PVP	43 ± 6	13 ± 7
10kDa PVP	41 ± 3	12 ± 6
55kDa PVP	50 ± 5	11 ± 6
1kDa PEGSH	45 ± 5	14 ± 5
5kDa PEGSH	49 ± 5	14 ± 6
SDS	42 ± 5	15 ± 4
MUA	44 ± 5	12 ± 4
MDA	45 ± 4	13 ± 4
MBA	44 ± 5	9 ± 3
TOAB	47 ± 6	8 ± 4
Ligand Exchanged TMOAB	48 ± 5	8 ± 3
As-synthesized TMOAB	28 ± 3	7 ± 2

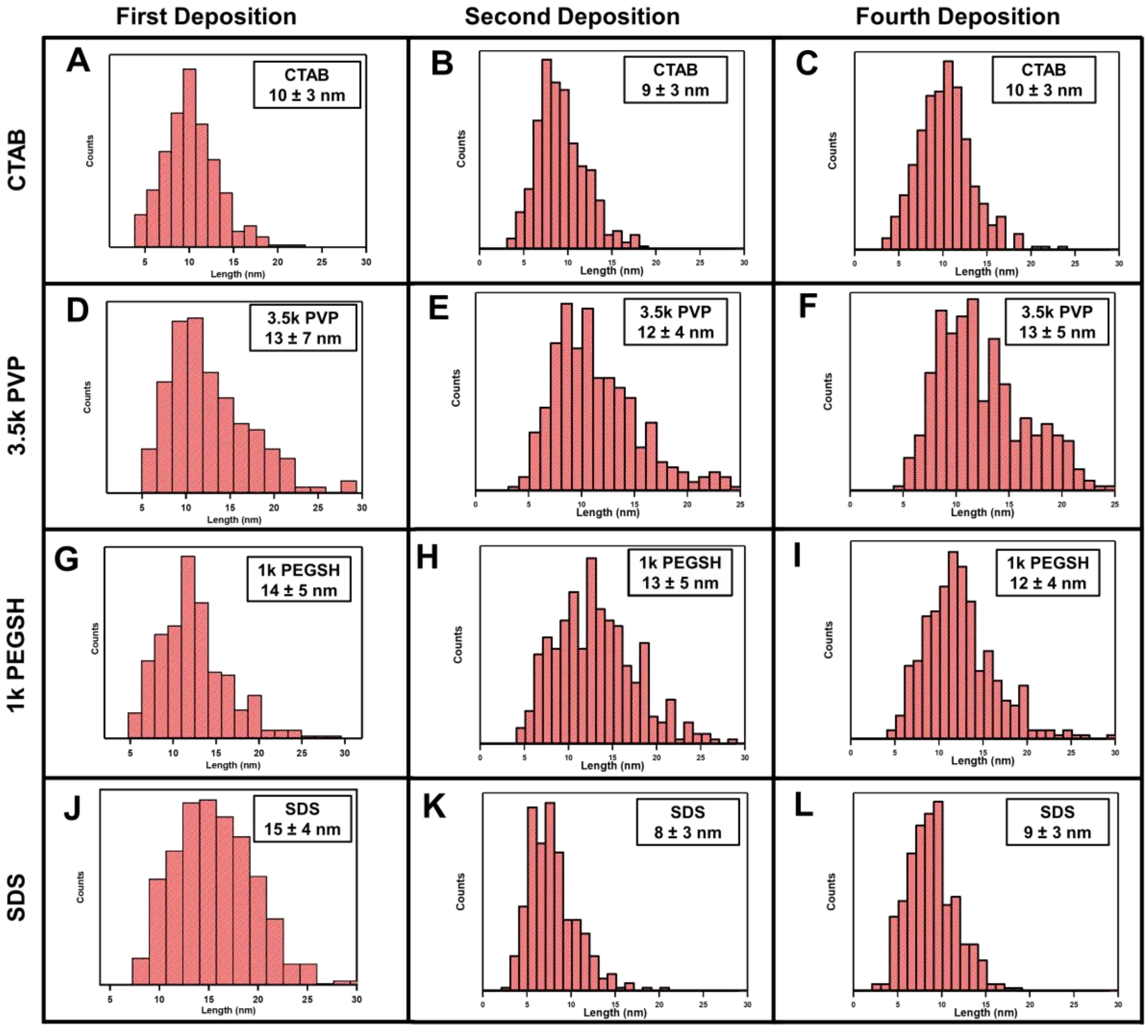

Fig S14. Histograms of size distributions of gold islandic deposition length from sequential deposition on $Cu_{2-x}Se$ capped with (A,B,C) CTAB (D,E,F) 3.5kDa PVP (G,H,I) 1kDa PEGSH and (J,K,L) SDS.

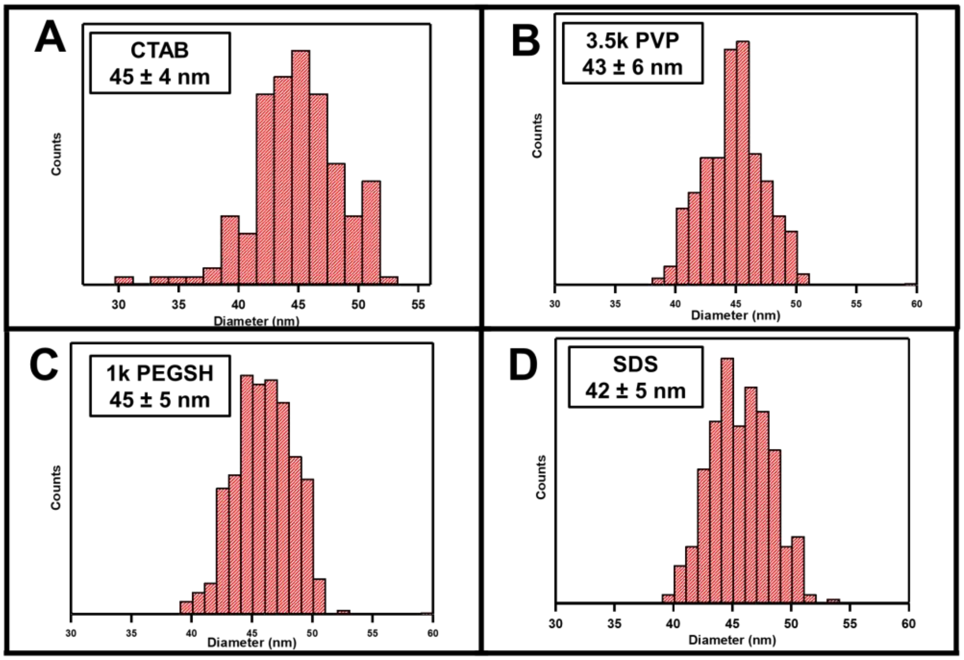

Figure S15. Histograms of size distributions of (A) as-synthesized CTAB capped $Cu_{2-x}Se$ and ligand exchanged $Cu_{2-x}Se$ with (B) 3.5kDa PVP, (C) 1kDa PEGSH and (D) SDS.

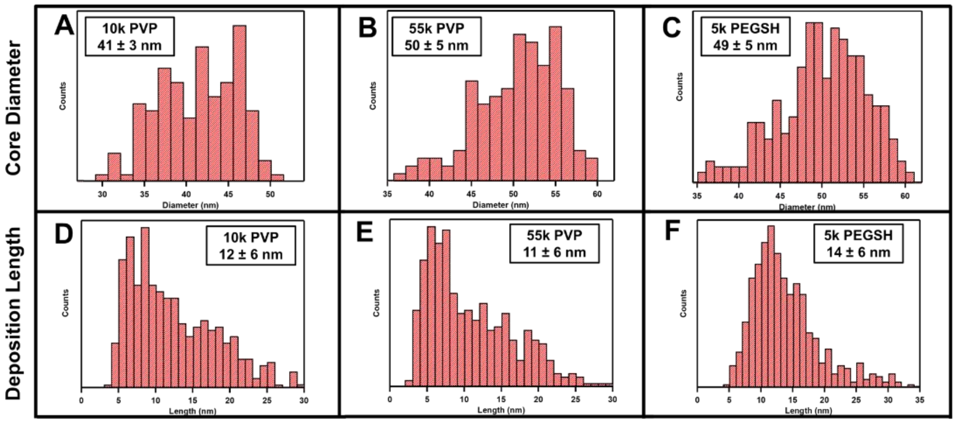

Figure S16. Histograms of size distributions of ligand exchanged $Cu_{2-x}Se$ and subsequent gold islandic deposition length on $Cu_{2-x}Se$ capped with (A,D) 10kDa PVP (B,E) 55kDa PVP, and (C,F) 5kDa PEGSH.

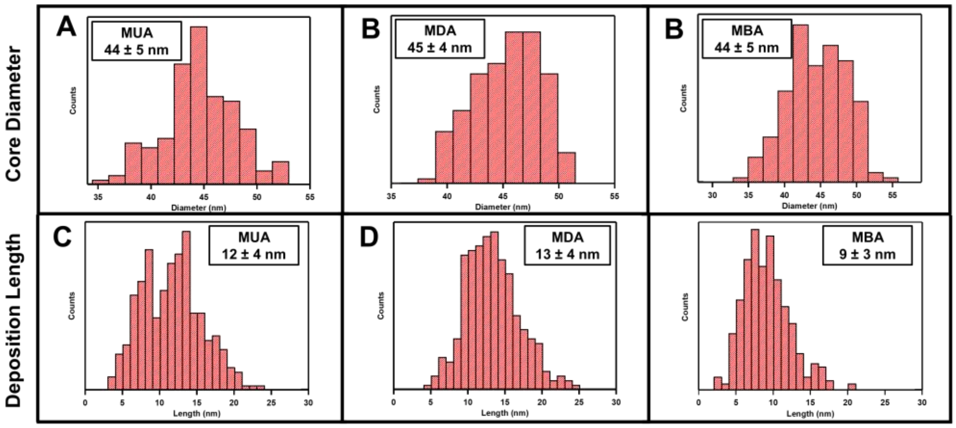

Figure S17. Histograms of size distributions of ligand exchanged $Cu_{2-x}Se$ and subsequent gold islandic deposition length on $Cu_{2-x}Se$ capped with (A) MUA (B) MDA, and (C) MBA.

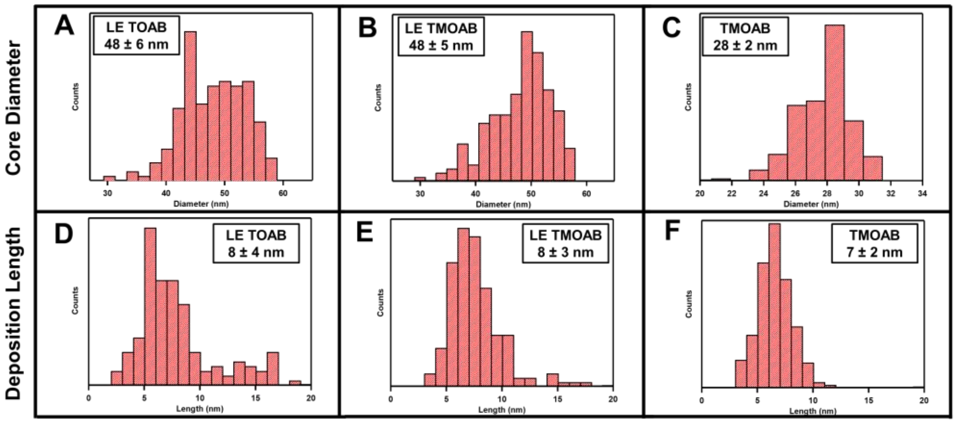

Figure S18. Histograms of size distributions and subsequent gold islandic deposition length on $Cu_{2-x}Se$ capped with (A,D) TOAB (ligand exchanged) (B,E) TMOAB (ligand exchanged), and (C,F) TMOAB (as-synthesized).

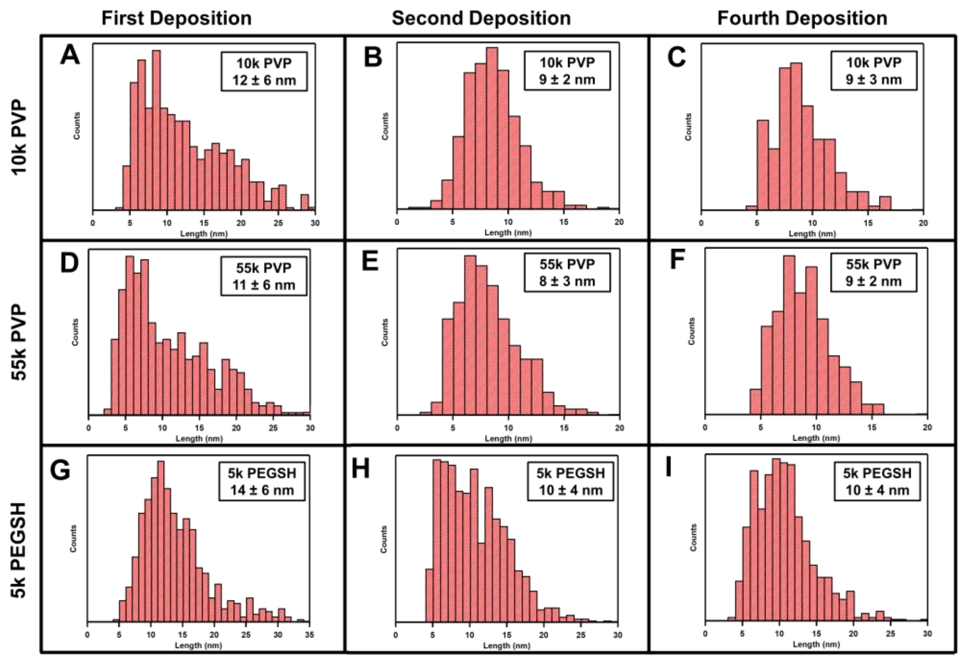

Figure S19. Histograms of size distributions of gold islandic deposition length from sequential deposition on $Cu_{2-x}Se$ capped with (A,B,C) 10kDa PVP (D,E,F) 55kDa PVP and (G,H,I) 5kDa PEGSH.

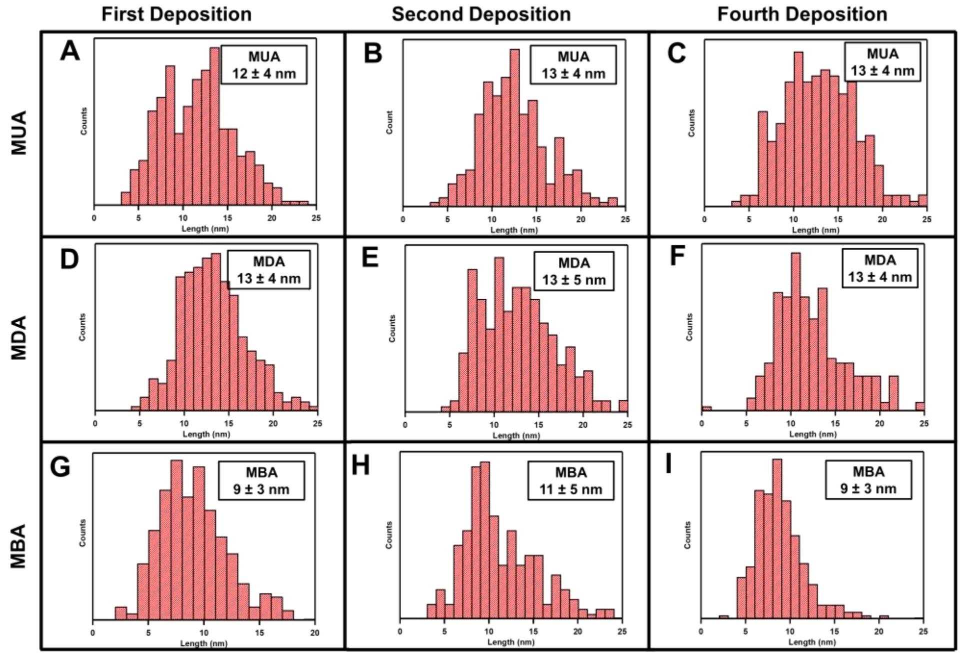

Figure S20. Histograms of size distributions of gold islandic deposition length from sequential deposition on $Cu_{2-x}Se$ capped with (A,B,C) MUA (D,E,F) MDA and (G,H,I) MBA.

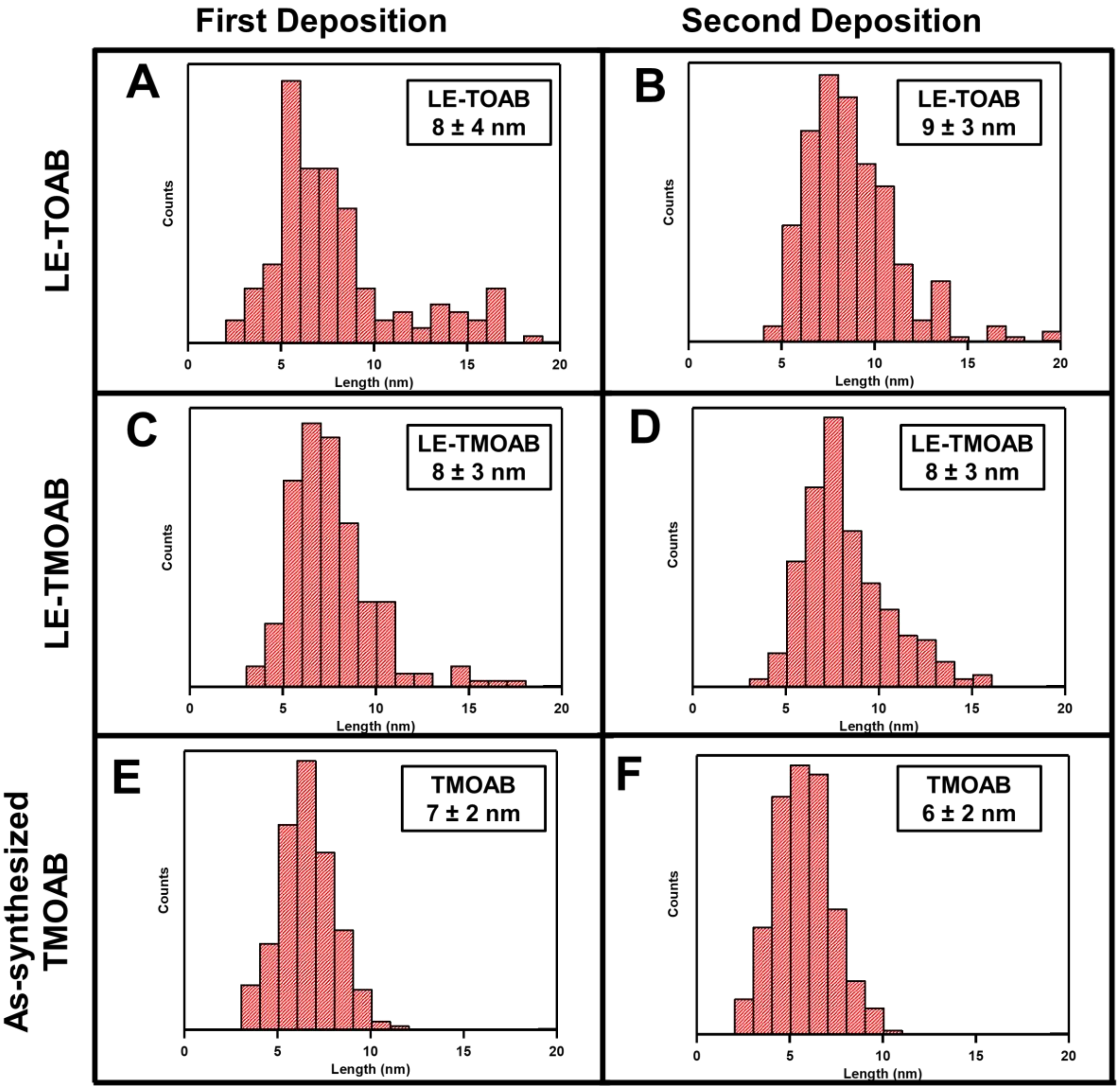

Figure S21. Histograms of size distributions of gold islandic deposition length from sequential deposition on $Cu_{2-x}Se$ capped with (A,B) TOAB (ligand exchanged) (C,D) TMOAB (ligand exchanged) and (E,F) TMOAB (as-synthesized).

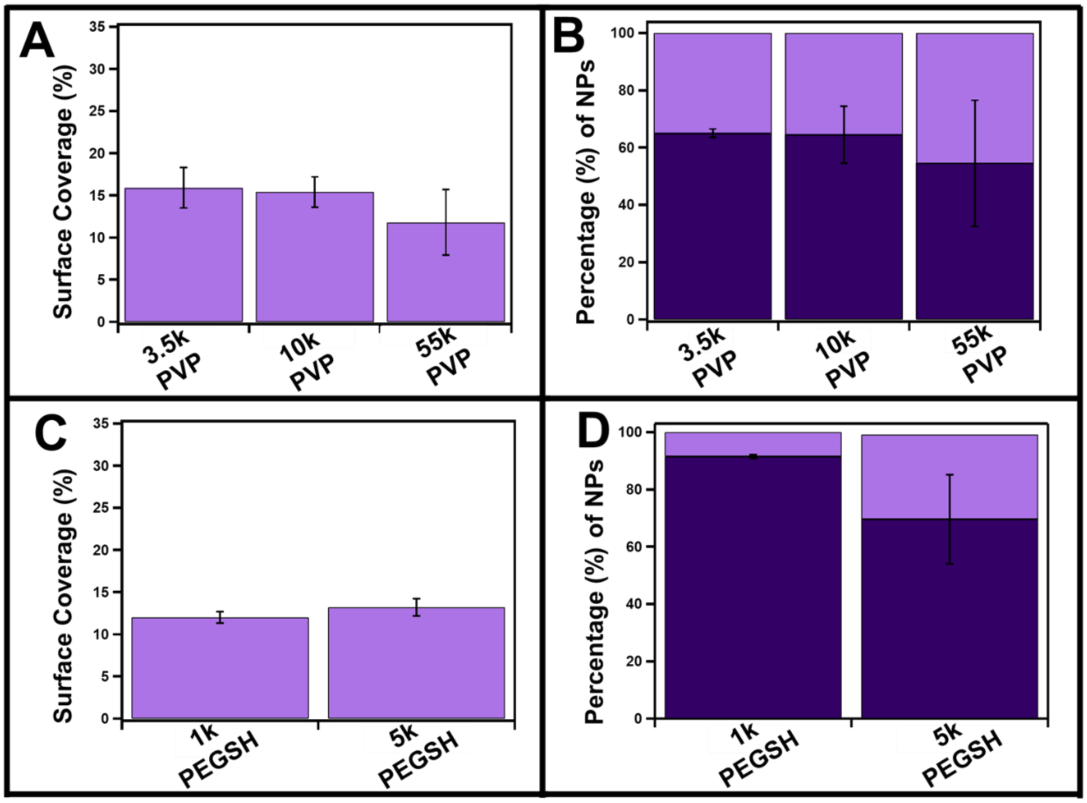

Figure S22. Percent surface coverage and percent modification of gold metal deposition on $Cu_{2-x}Se$ NPs capped with (A,B) PVP and (C,D) PEGSH.

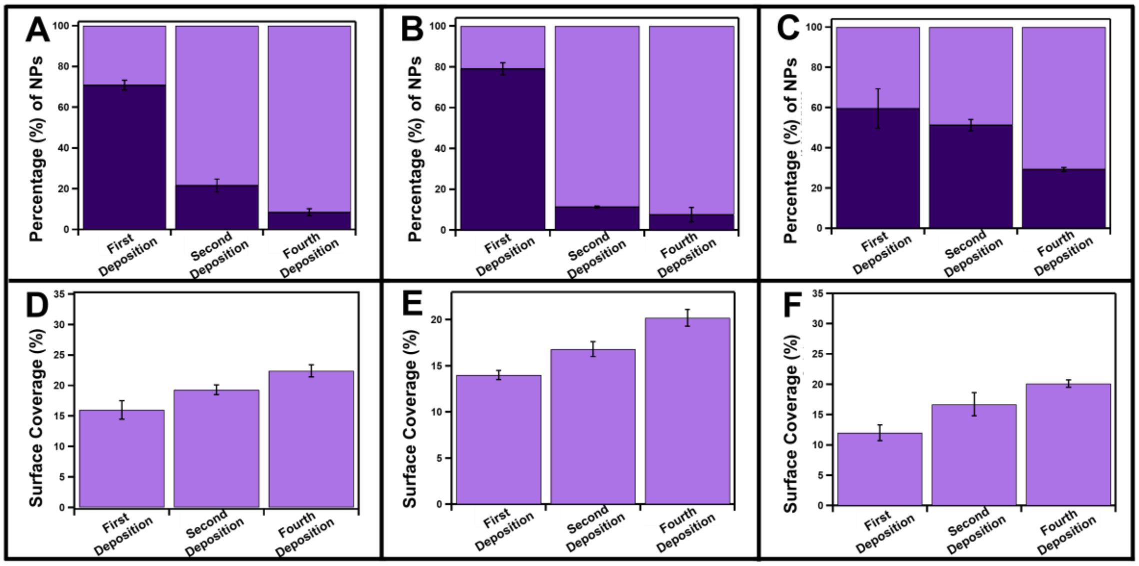

Figure S23. Percent modification and surface coverage of sequential gold metal deposition on $Cu_{2-x}Se$ NPs capped with (A,D) 55kDa PVP, (B,E) 10kDa PVP, and (C,F) 5kDa PEGSH for sequential depositions.

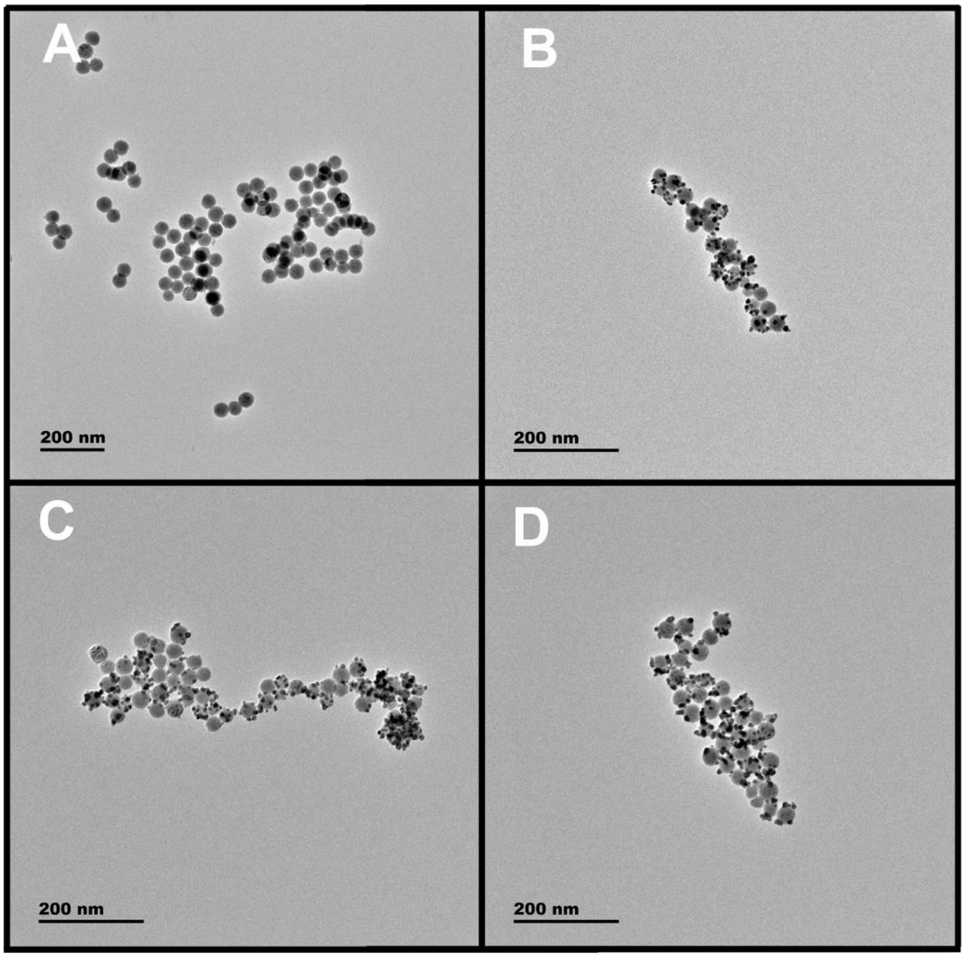

Figure S24. Representative TEM images of the $Cu_{2-x}Se$ (A) and first (B), second, (C) and fourth (D) sequential gold deposition on $Cu_{2-x}Se$ NPs capped with 10kDa PVP.

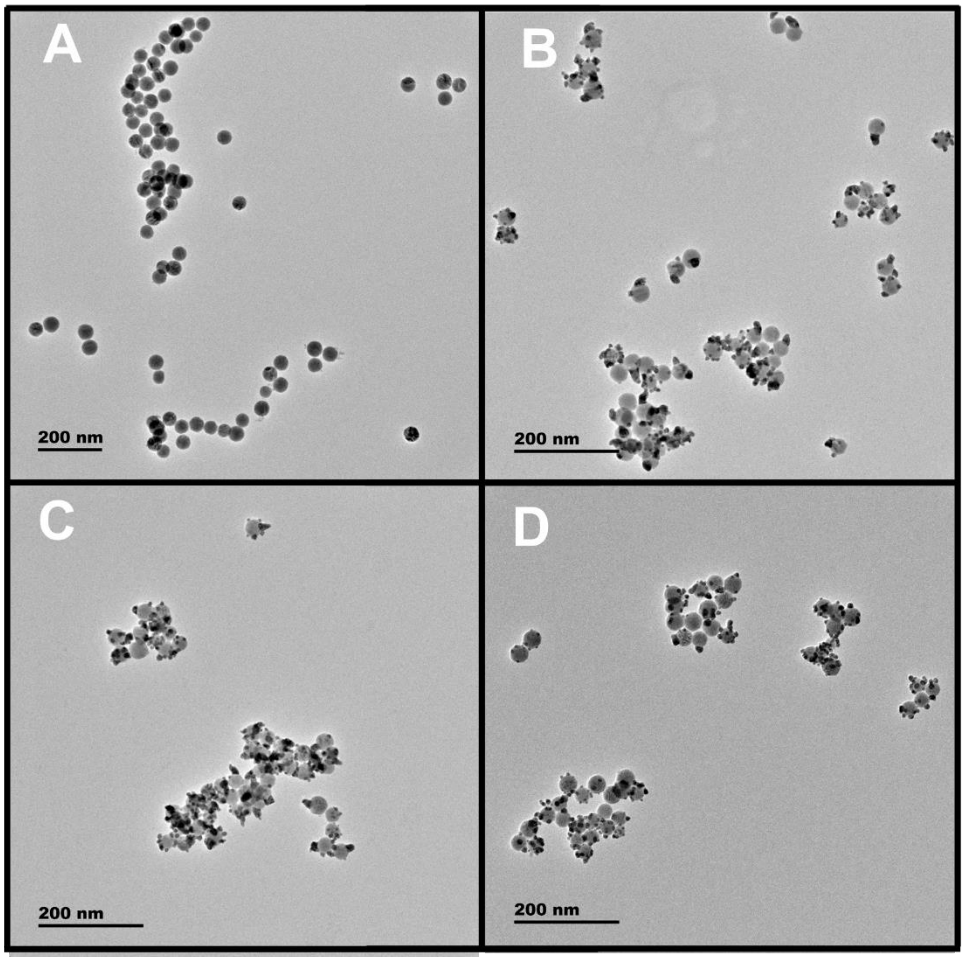

Figure S25. Representative TEM images of the $Cu_{2-x}Se$ (A) and first (B), second, (C) and fourth (D) sequential gold deposition on $Cu_{2-x}Se$ NPs capped with 55kDa PVP.

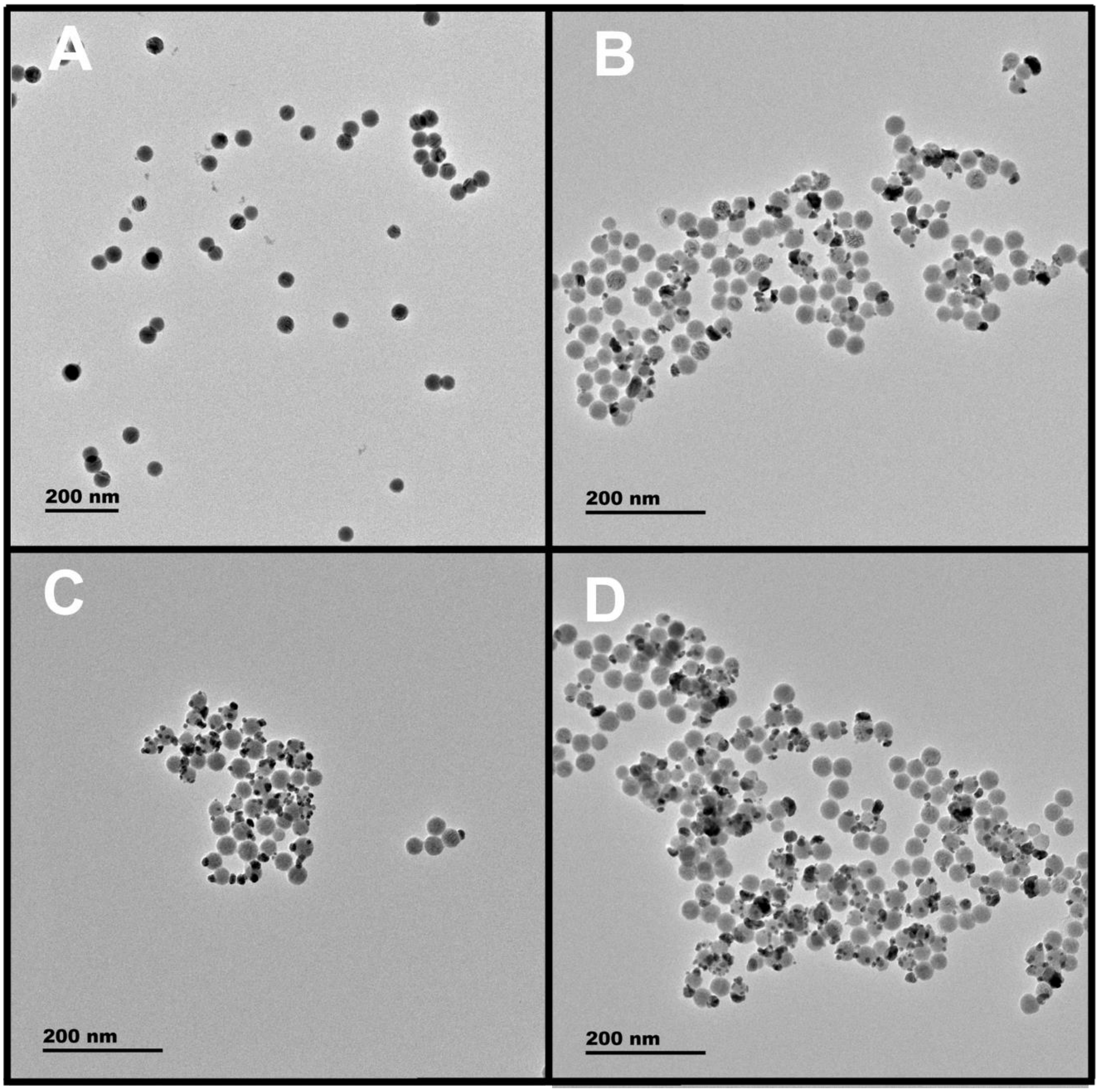

Figure S26. Representative TEM images of the $Cu_{2-x}Se$ (A) and first (B), second, (C) and fourth (D) sequential gold deposition on $Cu_{2-x}Se$ NPs capped with 5kDa PEGSH.

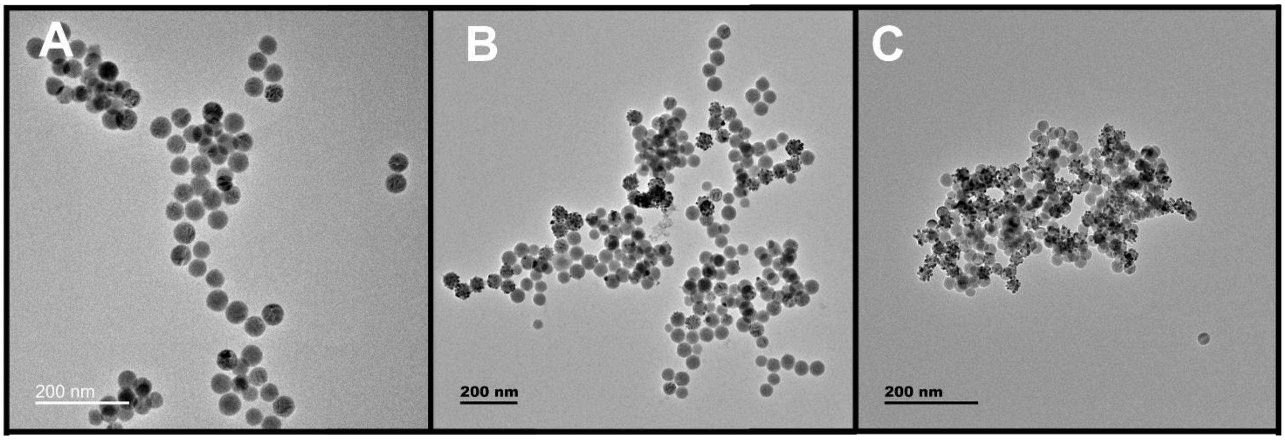

Figure S27. Representative TEM images of the $Cu_{2-x}Se$ (A) and first (B), and second (C) sequential gold deposition on $Cu_{2-x}Se$ NPs ligand exchanged with TMOAB.

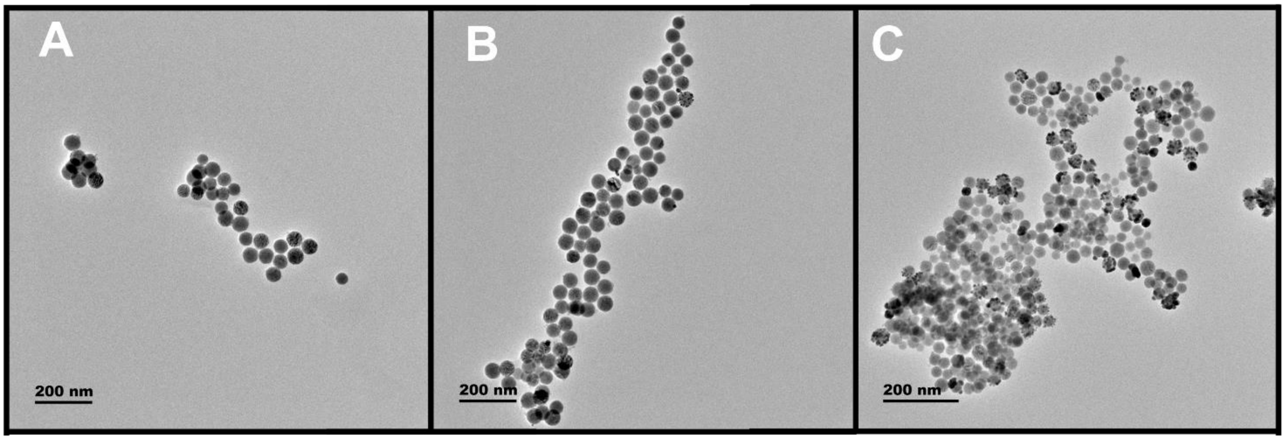

Figure S28. Representative TEM images of the $Cu_{2-x}Se$ (A) and first (B), and second (C) sequential gold deposition on $Cu_{2-x}Se$ NPs ligand exchanged with TOAB.

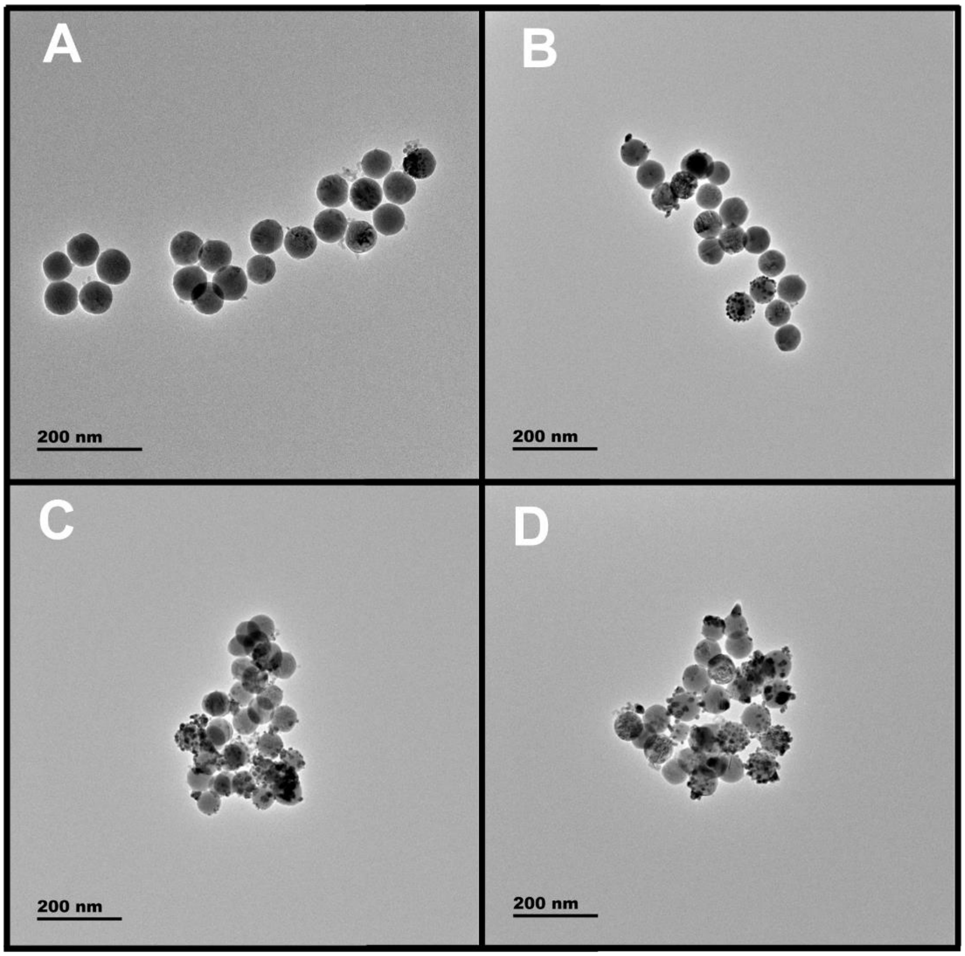

Figure S29. Representative TEM images of the $Cu_{2-x}Se$ (A) and first (B), second (C), and fourth (D) sequential gold deposition on $Cu_{2-x}Se$ NPs capped with MBA.

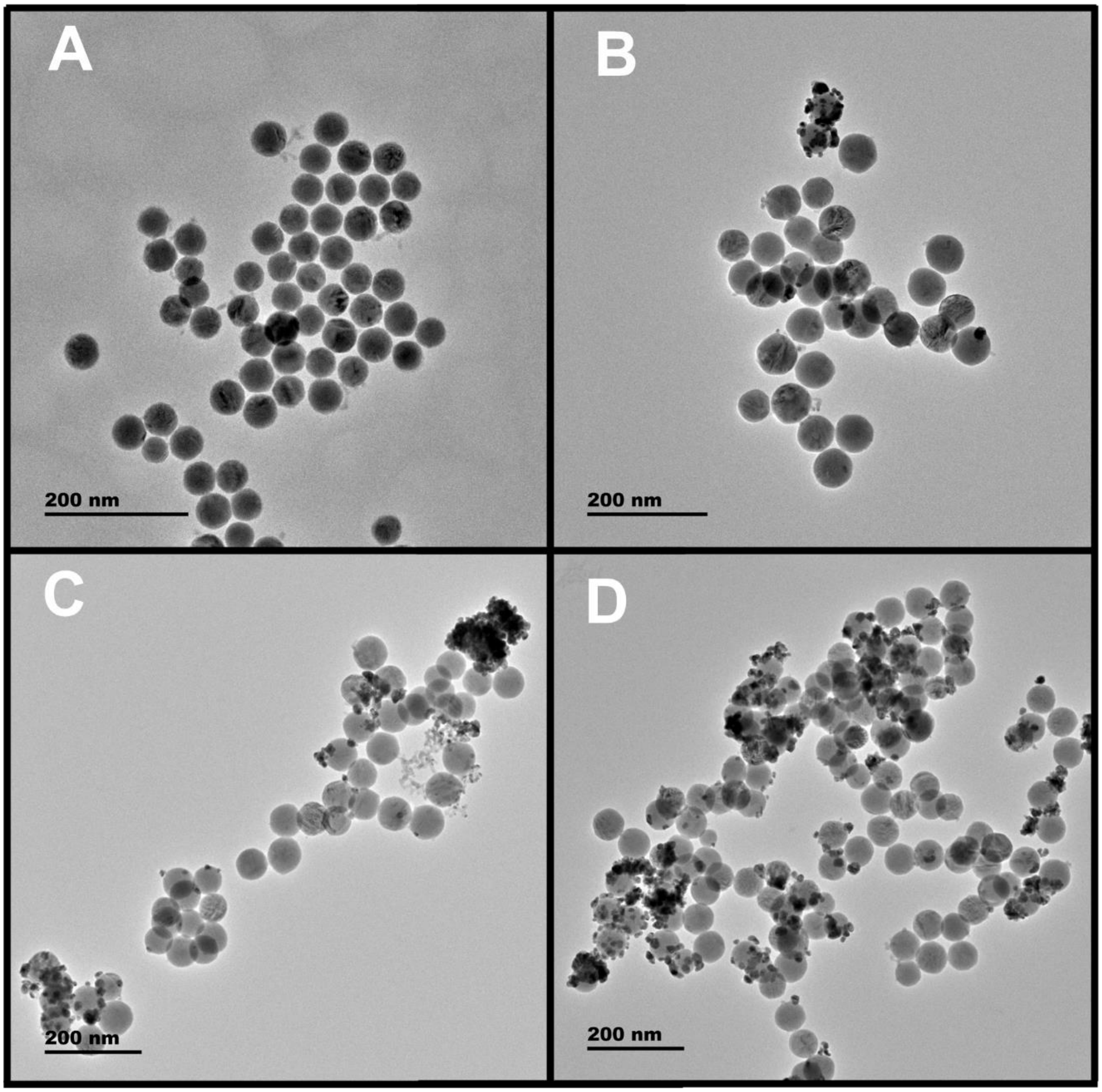

Figure S30. Representative TEM images of the $Cu_{2-x}Se$ (A) and first (B), second (C), and fourth (D) sequential gold deposition on $Cu_{2-x}Se$ NPs capped with MDA.

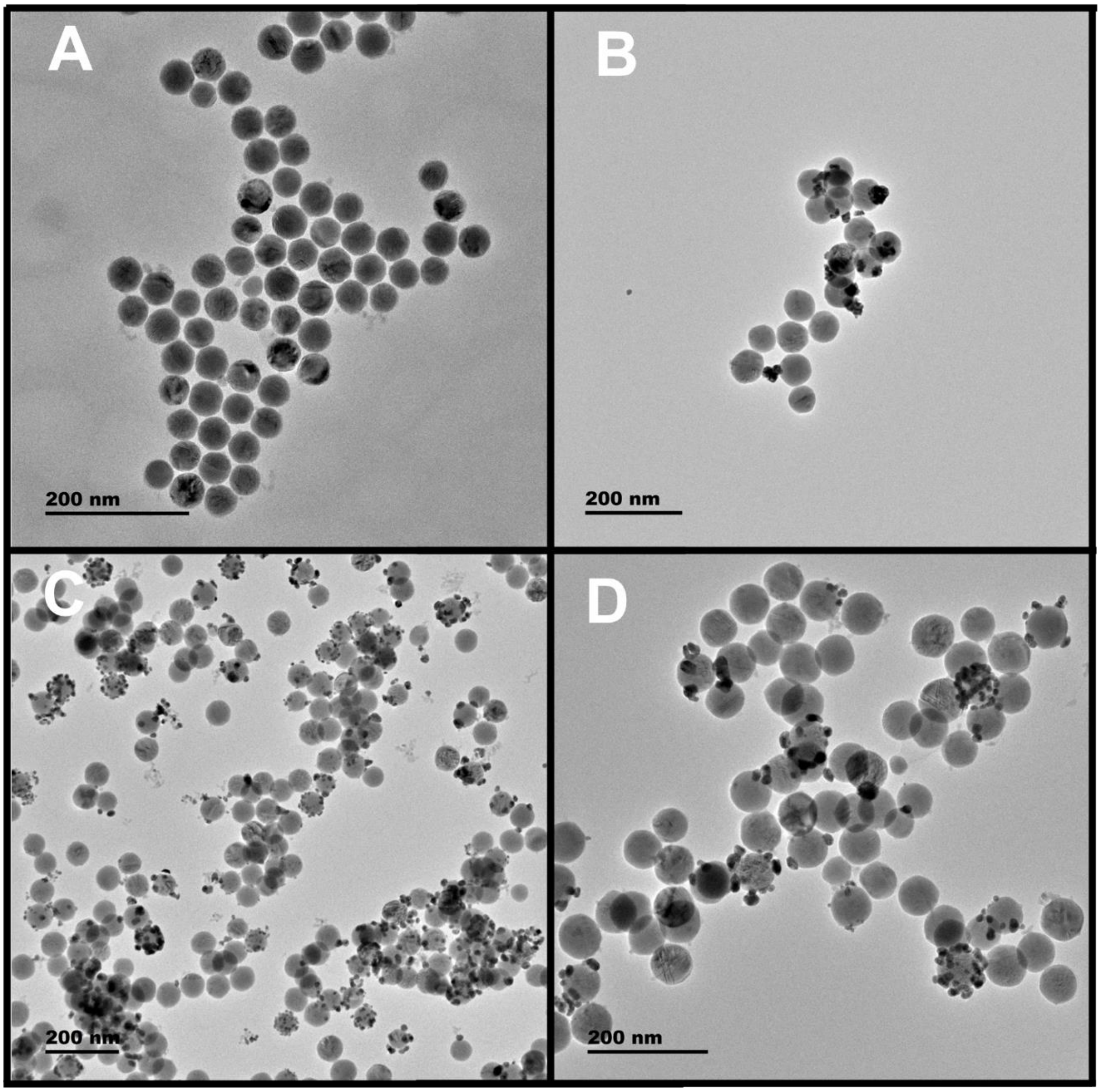

Figure S31. Representative TEM images of the $Cu_{2-x}Se$ (A) and first (B), second (C), and fourth (D) sequential gold deposition on $Cu_{2-x}Se$ NPs capped with MUA.

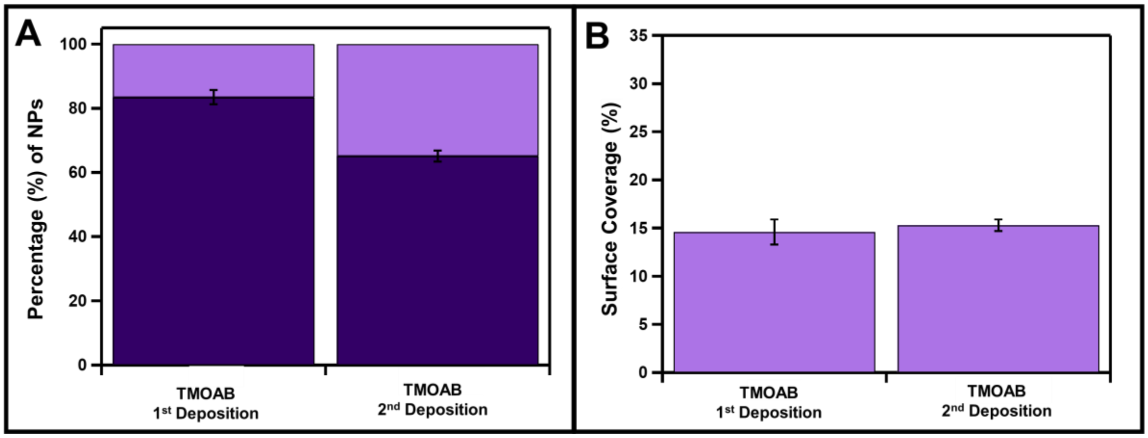

Figure S32. Comparative (A) percent modification and (B) surface coverage of $Cu_{2-x}Se$ functionalized with TMOAB (ligand exchanged from CTAB).

References

1. Smith, A. M.; Marbella, L. E.; Johnston, K. A.; Hartmann, M. J.; Crawford, S. E.; Kozycz, L. M.; Seferos, D. S.; Millstone, J. E., Quantitative Analysis of Thiolated Ligand Exchange on Gold Nanoparticles Monitored by ^{1}H NMR Spectroscopy. *Analytical Chemistry* **2015,** *87* (5), 2771-2778.
2. Bertini, I.; Turano, P.; Vila, A. J., Nuclear magnetic resonance of paramagnetic metalloproteins. *Chemical reviews* **1993,** *93* (8), 2833-2932.
3. Hartley, C. L.; Kessler, M. L.; Dempsey, J. L., Molecular-Level Insight into Semiconductor Nanocrystal Surfaces. *Journal of the American Chemical Society* **2021,** *143* (3), 1251-1266.
4. Smith, A. M.; Johnston, K. A.; Crawford, S. E.; Marbella, L. E.; Millstone, J. E., Ligand Density Quantification on Colloidal Inorganic Nanoparticles. *Analyst* **2017,** *142* (1), 11-29.
5. Knauf, R. R.; Lennox, J. C.; Dempsey, J. L., Quantifying Ligand Exchange Reactions at CdSe Nanocrystal Surfaces. *Chemistry of Materials* **2016,** *28* (13), 4762-4770.
6. De Roo, J.; Yazdani, N.; Drijvers, E.; Lauria, A.; Maes, J.; Owen, J. S.; Van Driessche, I.; Niederberger, M.; Wood, V.; Martins, J. C.; Infante, I.; Hens, Z., Probing Solvent–Ligand Interactions in Colloidal Nanocrystals by the NMR Line Broadening. *Chemistry of Materials* **2018,** *30* (15), 5485-5492.
7. Drijvers, E.; De Roo, J.; Martins, J. C.; Infante, I.; Hens, Z., Ligand Displacement Exposes Binding Site Heterogeneity on CdSe Nanocrystal Surfaces. *Chemistry of Materials* **2018,** *30* (3), 1178-1186.
8. Oliva-Puigdomènech, A.; De Roo, J.; Kuhs, J.; Detavernier, C.; Martins, J. C.; Hens, Z., Ligand Binding to Copper Nanocrystals: Amines and Carboxylic Acids and the Role of Surface Oxides. *Chemistry of Materials* **2019,** *31* (6), 2058-2067.
9. Dümbgen, K. C.; Leemans, J.; De Roo, V.; Minjauw, M.; Detavernier, C.; Hens, Z., Surface Chemistry of InP Quantum Dots, Amine–Halide Co-Passivation, and Binding of Z-Type Ligands. *Chemistry of Materials* **2023,** *35* (3), 1037-1046.

10. Anderson, N. C.; Hendricks, M. P.; Choi, J. J.; Owen, J. S., Ligand Exchange and the Stoichiometry of Metal Chalcogenide Nanocrystals: Spectroscopic Observation of Facile Metal-Carboxylate Displacement and Binding. *Journal of the American Chemical Society* **2013,** *135* (49), 18536-18548.
11. Crawford, S. E.; Andolina, C. M.; Smith, A. M.; Marbella, L. E.; Johnston, K. A.; Straney, P. J.; Hartmann, M. J.; Millstone, J. E., Ligand-Mediated “Turn On,” High Quantum Yield Near-Infrared Emission in Small Gold Nanoparticles. *Journal of the American Chemical Society* **2015,** *137* (45), 14423-14429.
12. Clogston, J. D.; Patri, A. K., Zeta Potential Measurement. In *Characterization of Nanoparticles Intended for Drug Delivery*, McNeil, S. E., Ed. Humana Press: Totowa, NJ, 2011; pp 63-70.
13. Yang, J.; Zhu, P.; Meng, F.; Guo, Q.; He, T.; Yang, Z.; Qu, W.; Li, H., Charge distribution modulation and morphology controlling of copper selenide for an enhanced elemental mercury adsorption activity in flue gas. *Chemical Engineering Journal* **2022,** *442*, 136145.
14. Xiao, Y.; Zhao, X.; Wang, X.; Su, D.; Bai, S.; Chen, W.; Fang, S.; Zhou, L.; Cheng, H.-M.; Li, F., A Nanosheet Array of Cu2Se Intercalation Compound with Expanded Interlayer Space for Sodium Ion Storage. *Advanced Energy Materials* **2020,** *10* (25), 2000666.
15. Yu, X., The preparation and characterization of cetyltrimethylammonium intercalated muscovite. *Microporous and Mesoporous Materials* **2007,** *98* (1), 70-79.
16. Chen, X. Q.; Li, Z.; Dou, S. X., Ambient Facile Synthesis of Gram-Scale Copper Selenide Nanostructures from Commercial Copper and Selenium Powder. *ACS Applied Materials & Interfaces* **2015,** *7* (24), 13295-13302.
17. Biesinger, M. C.; Hart, B. R.; Polack, R.; Kobe, B. A.; Smart, R. S. C., Analysis of mineral surface chemistry in flotation separation using imaging XPS. *Minerals Engineering* **2007,** *20* (2), 152-162.
18. Lu, N.; Zhang, M.; Jing, X.; Zhang, P.; Zhu, Y.; Zhang, Z., Electrospun semiconductor-based nano-heterostructures for photocatalytic energy conversion and environmental remediation: opportunities and challenges. *Energy & Environmental Materials* **2023,** *6* (2), e12338.
19. Rasheed, M.; Saira, F.; Batool, Z.; khan, H. M.; Yaseen, J.; Arshad, M.; Kalsoom, A.; Ahmed, H. E.; Ashiq, M. N., Facile synthesis of a CuSe/PVP nanocomposite for ultrasensitive non-enzymatic glucose biosensing. *RSC Advances* **2023,** *13* (38), 26755-26765.
20. Terlingen, J. G. A.; Feijen, J.; Hoffman, A. S., Immobilization of Surface Active Compounds on Polymer Supports Using Glow Discharge Processes: 1. Sodium Dodecyl Sulfate on Poly(propylene). *Journal of Colloid and Interface Science* **1993,** *155* (1), 55-65.
21. Karlsson, P. M.; Palmqvist, A. E. C.; Holmberg, K., Adsorption of Sodium Dodecyl Sulfate and Sodium Dodecyl Phosphate on Aluminum, Studied by QCM-D, XPS, and AAS. *Langmuir* **2008,** *24* (23), 13414-13419.
22. Kao, L. S.; Green, C. E., Analysis of Variance: Is There a Difference in Means and What Does It Mean? *Journal of Surgical Research* **2008,** *144* (1), 158-170.
